# Polymicrobial Sepsis-Induced Changes in Hepatic Stellate Cell Communication in Male C57BL/6J Mice

**DOI:** 10.3390/cells15110968

**Published:** 2026-05-24

**Authors:** Steven Timmermans, Céline Van Dender, Maxime Roes, Elise Moens, Tineke Vanderhaeghen, Jolien Vandewalle, Claude Libert

**Affiliations:** 1 Center for Inflammation Research, Vlaams Instituut voor Biotechnologie (VIB), 9052 Ghent, Belgium; steven.timmermans@irc.vib-ugent.be (S.T.); celine.vandender@irc.vib-ugent.be (C.V.D.); maxime.roes@irc.vib-ugent.be (M.R.); elise.moens@irc.vib-ugent.be (E.M.); tineke.vanderhaeghen@irc.vib-ugent.be (T.V.); jolien.vandewalle@irc.vib-ugent.be (J.V.); 2 Department of Biomedical Molecular Biology, Ghent University, 9000 Ghent, Belgium

**Keywords:** hepatic stellate cell, sepsis, cell-cell communication

## Abstract

Sepsis, which affects 49 million people yearly, killing 11 million of them, is known to induce severe liver dysfunction. It is characterized by extensive metabolic reprogramming, resulting in acute metabolic loss of function and maladaptive repair that can prime the organ for fibrosis rather than functional regeneration. To understand how intercellular communication dictates these outcomes, we performed cell type-specific bulk RNA-sequencing on hepatocytes (HEP), hepatic stellate cells (HSCs), liver sinusoidal endothelial cells (LSECs), Kupffer cells (KC), and CD45^+^ leukocytes (CD45) from mice following polymicrobial sepsis. Cell-cell communication analyses using CellChat and NicheNet revealed a clear reorganization of the hepatic environment. While HSCs remain largely quiescent during homeostasis, after sepsis, they become the liver’s central signaling hub and broadcast potent fibrogenic and chemotactic signals (e.g., Ccl7) to surrounding cells. This actively suppresses hepatocyte metabolic functions, promotes leukocyte infiltration, and may further initiate early fibrogenic priming. Our findings highlight HSCs as regulators during septic acute liver injury, revealing communication nodes that could be targeted to constrain fibrosis responses and promote normal functions and repair.

## 1. Introduction

The liver is a central and indispensable organ in maintaining systemic homeostasis in mammals and all other vertebrate animals. It plays a central role in immune regulation processes [1,2], production of blood plasma proteins such as albumin and blood clotting factors, bile acid metabolism, and overall metabolism (e.g., lipid metabolism, gluconeogenesis, ketogenesis) [2]. It is also the primary organ responsible for detoxification of toxins and other foreign substances. Through the portal veins, the liver is directly exposed to products coming from the intestine, including nutrients, xenobiotics, bacteria and bacterial products, and metabolites [2,3]. This complex interplay of functions and environment has led to a distinct spatial organization in the organ, called zonation, where the functions of the cells depend not only on the cell type but also on their location within the organ and the smaller units of the organ, the sinusoids [1,4].

The complete set of the liver’s functions and responses is provided by a complex interplay between multiple cell types that compose the organ. Most metabolic functions are performed by hepatocytes (HEPs), the primary liver parenchymal cells, which account for about 60% of the liver cell population and 80% of the liver mass [5,6]. The other non-parenchymal cells are involved in structural support, immune surveillance, and tissue repair. Liver sinusoidal endothelial cells (LSECs) form the lining of the smallest blood vessels in the liver. Unlike most other endothelial cells, they form a highly fenestrated barrier and function as a filtering and scavenging cell population that regulates vascular tone and leukocyte trafficking. Together with hepatocytes, they delineate the space of Disse, which is filled with blood plasma and forms the residence of the hepatic stellate cells (HSCs) [7,8]. The HSCs are a subset of fibroblasts and are quiescent under homeostatic conditions and provide the liver’s storage of vitamin A. Furthermore, they secrete niche growth factors and are responsible for extracellular matrix (ECM) maintenance [9,10,11]. Upon liver injury, HSCs become activated and contribute to liver remodeling and repair. However, this activation can have detrimental consequences and represents the main pathway leading to collagen deposition, which may result in liver fibrosis and, in extreme cases, liver cirrhosis. Increasing evidence points to a role for HSCs in immunomodulatory processes in the liver, such as regulating T-cell activity or antigen presentation, providing further evidence of the highly complex interplay between liver cell types [11,12,13]. Finally, the Kupfer cells (KCs) make up the residential immune compartment of the liver. They are tissue-resident macrophages that have the primary function to clear apoptotic bodies and cellular debris, as well as to remove pathogens delivered via the portal circulation [14,15].

Sepsis is defined as a potentially lethal, dysregulated systemic host response to infection. It affects 49 million people, yearly killing 11 million of them. Sepsis is a systemic pathology that involves profound reprogramming of most physiological pathways in the liver [16,17]. Cecal ligation and puncture (CLP) is considered the gold standard mouse model for polymicrobial sepsis [18]. As described by us and others [16,17,19], the liver undergoes extensive molecular and cellular changes as part of the systemic inflammatory response that is associated with sepsis, whereby the inflammatory state directly alters cell functions of the liver cell populations. Hepatocytes initially switch to the acute phase response, presumably at the cost of other functions, such as systemic metabolic support [20]. The immune part of the response is driven primarily by KCs and LSECs, which attract other immune cells from the CD45^+^ circulating pool. It is well established that HSCs are activated in sepsis, interact with KCs, and thereby contribute to the immune response through a positive feedback loop that also initiates liver remodeling, ECM remodeling and collagen deposition [21], which in turn further signals the behavior of hepatocytes. Circulating immune cells migrate to and extravasate into the liver in response to bacteria and other chemoattractant molecules, such as cytokines produced by liver cells, as mentioned before [22]. These infiltrating cells are vital for combating ongoing infection, but their actions will also contribute to liver cellular stress and cause significant collateral damage [22]. Overall, sepsis leads to significant alterations and progressive decline of physiological liver function, with some evidence for long-term liver dysfunction and sensitization to fibrotic disease [17,19].

The involvement of the liver as a primary organ in sepsis pathology has been well established. On the one hand, the liver is directly involved in early pathology (e.g., acute phase response). On the other hand, liver failure is a significant contributing factor to sepsis mortality, an observation known as sepsis-associated liver dysfunction. Research of the liver during sepsis, e.g., by RNA sequencing (RNA-seq) studies, so far has relied primarily on bulk whole tissue data, which has revealed metabolic disturbances resulting from the failure of key transcription factors such as peroxisome proliferator-activated receptor-alpha (PPARα, Ppara) [23], glucocorticoid receptor (GR) [24], and hepatocyte nuclear factor-4 alpha (HNF4α) [25], as well as the induction of an acute phase response contributing to liver regeneration. Although some cell type-specific differences have been described previously, the question about the extent to which intercellular interactions shape these functional differences during sepsis remains largely unknown. This question is the focus of the present study. To this end, we isolated LSECs, hepatocytes, HSCs, KCs, and other CD45^+^ cells from the liver of mice, 8 h after sham or CLP surgery, and performed bulk RNA-Seq. We first analyzed global cell-cell communication using CellChat [26], from which we identified HSCs as a primary differential driver. This was followed by a more targeted analysis using CellChat and NicheNet [27]. We identify the ligands and receptors responsible for cell-cell communication in sepsis, which reveals new mechanistic insights and potential druggable targets.

## 2. Materials and Methods

### 2.1. Mice

Male C57BL/6J mice of 12 weeks old were purchased from Janvier. All mice were housed in individually ventilated cages at standard housing conditions (22 °C, 14/10 h light/dark cycle) with food (chow diet consisting of 18% proteins, 4.5% fibers, 4.5% fat, 6.3% ashes, Provimi Kliba SA, Kaiseraugst, Switzerland) and water ad libitum in a specific pathogen-free facility. Mice were acclimated for 7 days before surgery. All experiments were approved by the institutional ethics committee for animal welfare of the Faculty of Sciences, Ghent University, Belgium. All procedures were carried out in accordance with the relevant guidelines and regulations.

Mice were subjected to CLP operation [18] or the sham control procedure. Briefly, mice were anesthetized with isoflurane, followed by a 1 cm incision in the abdominal skin and ventral muscles. The cecum was exposed and ligated for 75% and punctured twice with a 21-gauge needle, which allows the cecal content to leak into the abdomen and to cause a systemic infection leading to sepsis. The abdominal musculature and skin were closed with simple running sutures and metallic clips, respectively. Sham mice underwent the same procedure without ligating and puncturing the cecum. No analgesics, antibiotics, or fluid resuscitation were administered during the short post-operative period prior to euthanasia, in order to avoid interference with the inflammatory process. Eight hours after sham or CLP surgery, mice (*n* = 4/group, 8 mice in total, mixed in 2 cages) were sacrificed with CO_2_, and livers were isolated to generate a single-cell suspension. All four CLP mice met the a priori determined inclusion criterion, defined as a detectable reduction in body temperature to 35 °C or lower. For the sham mice, we did not have inclusion/exclusion criteria. Humane endpoints were predefined, and none of the mice reached these endpoints. No a priori sample size calculation was performed, as this was an exploratory (pilot) study. Blinding was not implemented in this study. Investigators were aware of group allocation during all stages of the experiment, including allocation, experimental procedures, and data analysis.

### 2.2. Cell Separation and RNA-Seq

Hepatocytes, liver sinusoidal endothelial cells, hepatic stellate cells, Kupffer cells, and (other than KC) CD45^+^ cells were used. The isolation procedure was adapted from Bonnardel et al. [28]. Following retrograde cannulation, livers were first perfused with an EDTA-containing solution to remove blood, and then with a 0.4 mg/mL collagenase I-containing solution for 5 min (6 mL/min). The livers were minced and incubated for 20 min in 0.4 mg/mL collagenase I and 10 U/mL DNase at 37 °C. Subsequent steps were performed at 4 °C. Cell suspensions were filtered through a 100 µm mesh, centrifuged at 400 g for 7 min, and resuspended in 2 mL red blood cell lysis buffer for 2 min. Cells were washed with PBS, filtered through a 40 µm mesh, and centrifuged twice for 1 min at 50 g, yielding a hepatocyte-enriched fraction (pellet) and a leukocyte/LSEC/HSC-enriched fraction (supernatant). Both fractions were further centrifuged at 400 g for 7 min prior to FACS staining. Cells were pre-incubated with 2.4G2 antibody (553142, BD Biosciences, Franklin Lakes, NJ, USA) to block Fc receptors, and stained with CD31 (11-0311-81, Thermofisher, Waltham, MA, USA) and CD45 (25-0451-81, Thermofisher) antibodies (for the hepatocyte-enriched fraction) or CD31 (11-0311-81, Thermofisher), CD61 (104307, Biolegend, San Diego, CA, USA), PDGRb (13-1402-82, Thermofisher), CD45 (25-0451-81, Thermofisher), F4/80 and TIM4 (129907, Biolegend) antibodies (for the leukocyte/LSEC/HSC-enriched fraction) in the dark for 30 min at 4 °C. Cell viability was assessed using the Fixable Viability dye eFluor780 (65-0865-14, Thermofisher). Hepatocytes were sorted using a FACSDiscover S8 (BD Biosciences), while LSECs, HSCs, CD45 and KCs cells were sorted using a FACSAria^TM^ III (BD Biosciences).

These purified cell populations were used for standard bulk RNA-seq transcription profiling. One sample was lost during processing, leaving 4 replicates per condition (*n* = 4) except for the HSC_Sham group, where only 3 samples remained (*n* = 3). For each cell type and sample, a TruSeq stranded sequencing library was created and sequenced for 100 cycles on an Element AVITI sequencer (Element biosciences, San Diego, CA, USA). The reads were quality checked with fastqc and no specific preprocessing steps were required. Reads were mapped to the mouse reference transcriptome/genome (mm39/gencode v28) with STAR (2.7.10b) [29], and read counts were obtained during alignment using the STAR “--quantMode GeneCounts” option. Differential gene expressions were assessed with the DESeq2 [30] package (v 1.42.1), with the FDR set at 1%.

### 2.3. Analysis of Differentially Expressed Genes

Differentially expressed genes obtained with DESeq2 were used for transcriptional binding motif finding in the promoter using HOMER (v5.1), using the 1 kb upstream sequence. For functional enrichment analysis of KEGG pathways, we made use of the clusterProfiler (v4.20.0) R package [31,32]. Both functional over-representation tests and more complex gene set enrichment analyses were also performed with clusterProfiler, using statistically significant differentially expressed genes ranked by log2-fold change as input. All visualizations were created using included graphing functions or custom made with “ggplot2” (v4.0.3). Gene set overlaps were visualized as UpSet plots using “ggVennDiagram” (v1.5.7). All significance bars were added with “ggsignif” (v0.6.4) [33] as mean ± SD.

### 2.4. Cell-Cell Communication

The R packages CellChat (v2.1.2) [26,34] and NicheNet (2.2.0) [27] were used to investigate cell-cell communication between the 5 cell types in the context of CLP-induced differential gene expression. CellChat was used to obtain a global overview of changes in cell-cell communication interactions, i.e., who is talking to whom and how much. In the ranknet function, to obtain a list scored by relative information flow, CellChat uses the Wilcoxon test with Benjamini-Hochberg FDR correction. After CellChat analyses, NicheNet was used to zoom in on cell-cell communication, to find out what message(s) are being sent between the different cell types.

## 3. Results

### 3.1. Liver Cell Types in Mouse Sepsis

Bulk RNA-seq was performed on FACS-purified hepatocytes (HEP), liver sinusoidal endothelial cells (LSECs), hepatic stellate cells (HSCs), Kupffer cells (KCs) and non-KC CD45^+^ immune cells (CD45), which will include monocytes, monocyte-derived macrophages, T cells, B cells, dendritic cells, NK cells and neutrophils, 8 h after sham or CLP to investigate cell type-specific functional differences and cell-cell communication during sepsis.

**Figure 1:**
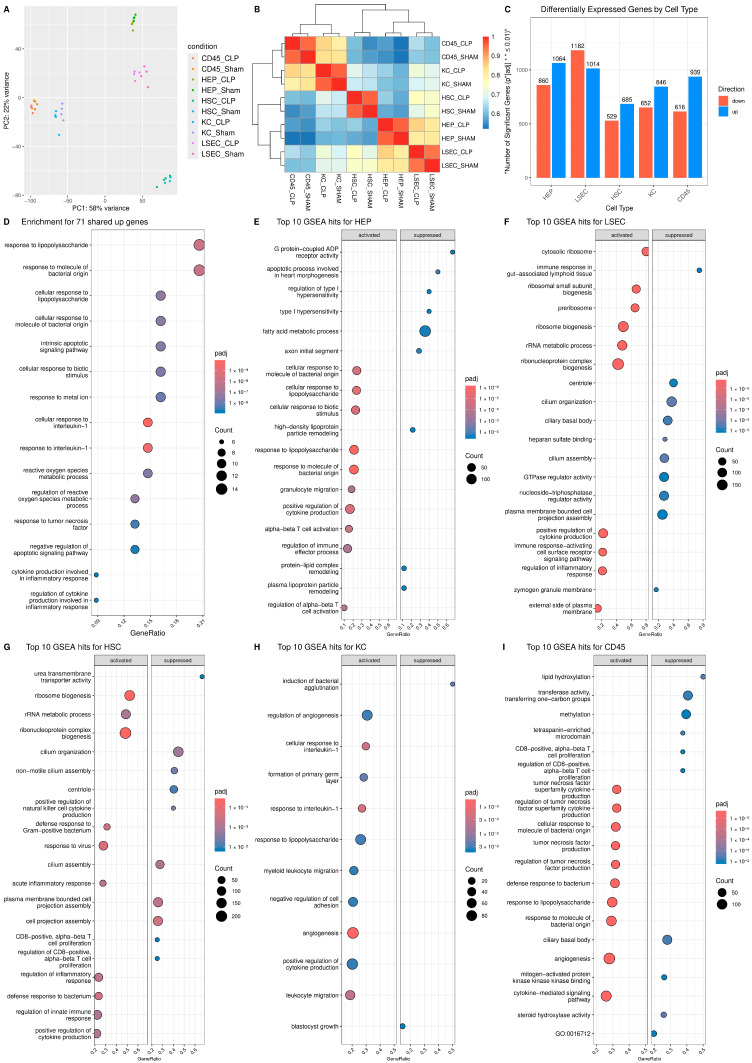
Quantitative interpretation of bulk RNA-seq data of five different cell types sorted from livers of sham-treated and CLP-treated mice. (**A**): PCA plot of the dataset; (**B**): Heatmap of samples, all genes (Pearson correlation). (**C**): Barplot of significantly up- and downregulated genes by cell type (*p* < 0.01). (**D**): Functional enrichment (GO) top 10 of 71 genes upregulated in all cell types. (**E**–**I**): top 10 activated and suppressed pathway results from Gene Set Enrichment Analysis (GSEA) of HEP (**E**), LSEC (**F**), HSC (**G**), KC (**H**) and CD45 (**I**). padj: FDR adjusted *p*-value; Count: circle size, number of genes matched to the term; GeneRatio: ratio of number of genes annotated to the term relative to the input set (1 = 100%, 0.5 = 50%, 0 = 0%).

The sorted cell types show clearly distinct expression profiles, with cell type being the primary driver of variation, with CLP/Sham only a distant second (Figure 1A,B). KCs and other CD45 cells cluster closely, which is consistent with their shared immune identity, as do LSECs and HEPs, despite these not having such shared roots or functions. The purity of the sorted populations was validated using the following cell type-specific markers: Albumin (*Alb*) for HEPs, Stabilin 2 (*Stab2*) for LSECs [8,35], lecithin-retinol acyltransferase (*Lrat*) for HSCs [36] and C-type lectin domain family 4 member F (*Clec4f*) for KCs [15] (Figure S1A–D). Most markers were uniquely expressed by the corresponding cell type, except *Alb*, which was also quite highly expressed in LSECs. This could suggest slight contamination, and/or it may explain the closer expression profile between LSECs and HEPs. However, during FACS sorting, larger cells were excluded by forward and side scatter, which is supposed to exclude mixing of HEPs with other cells and contraindicates sequencing of doublets or classification of HEPs as LSEC. However, LSECs may internalize HEP-derived vesicles containing mRNA, providing an alternative explanation for their similarity [37,38]. The CLP itself also affects the expression of several of these, but does not alter the specificity. Cholangiocytes were not included among the selected liver cell populations for practical reasons. Consistently, expression of the cholangiocyte marker Epithelial Cell Adhesion Molecule (*Epcam*) was low across all cell types (Figure S1E). To study differential gene expressions in the different cell types, as induced by sepsis (compared to sham conditions), we considered significant differences with an FDR set at 1% (=0.01). No LFC cut-off filter was applied unless otherwise specified. The number of differentially expressed genes (DEGs) upon sepsis varied by cell type, with LSECs showing the largest total set of DEGs and being the only cell type in which downregulated genes outnumbered upregulated genes, followed by HEPs (Figure 1C). HSCs showed the smallest overall response, with the fewest DEGs. Although some overlap was observed between cell types, no genes were oppositely regulated in any population. When studying the upregulated genes, we identified a core set of 71 genes that were consistently upregulated by CLP across all cell types, whereas no comparable set was observed for the downregulated genes (Figure S1J).

The shared genes were used for GO overrepresentation analysis, and the individual cell type DESeq2 results were subjected to gene set enrichment analysis with clusterprofiler. The shared genes show strong enrichment for pro-inflammatory signaling and cell death regulation (Figure 1D). All cell types, except LSECs, show clear activation of pro-inflammatory signaling pathways (e.g., response to LPS, TNF signaling, response to bacterium, etc.) as shown in Figure 1E–I, which is in line with the increased expression in pro-inflammatory markers, such as lipopolysaccharide binding protein (*Lbp*, Figure S1F), tumor necrosis factor (*Tnf*, Figure S1G). C-reactive binding protein (*Crp*, Figure S1H) is highly expressed but not significantly unregulated, which may be a Sham effect. For HEP specifically, we also see a strong suppression of G-protein receptor signaling and fatty acid and cholesterol metabolism, indicating a clear metabolic shift to acute phase reprogramming. The main lipid metabolism transcription factor *Ppara* is also significantly downregulated in these cells (LFC: −1.83, *p*-adj: 0.0036), directly supporting these findings. Both LSECs and HSCs showed a significant activation of ribosomal machinery, indicating upregulation of the protein production capacity in these cells (Figure 1F,G). LSECs furthermore show cilia hits (suppressed, Figure 1F). KCs show strong positive signals for migration, adhesion and cytokine production, while downregulated genes are associated with immune response and cell activation, suggesting their state is in a strong negative feedback loop attempting to limit activation (Figure S2G,H). KCs do not show any strong enrichment for phagocytosis, in contrast to other CD45 infiltrating cells, which show increased phagocytosis, which may indicate partial compensation (Figure S3A).

### 3.2. Global Cell-Cell Communication

**Figure 2:**
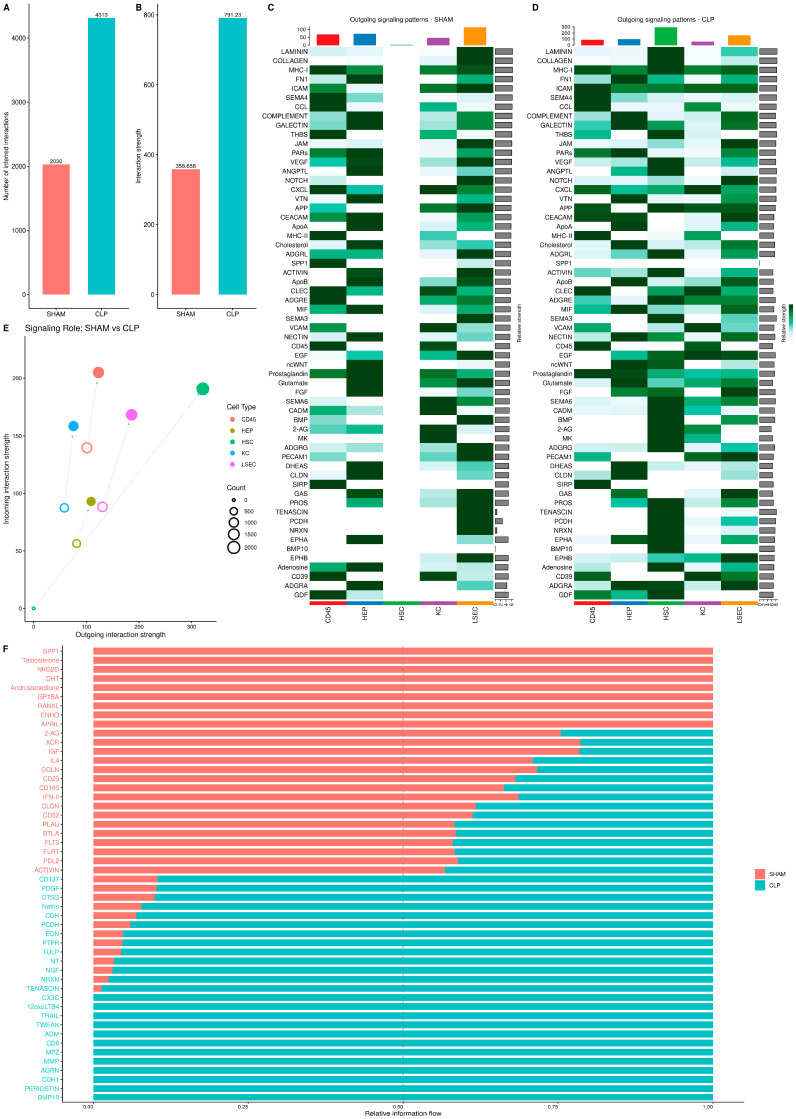
Liver cell-cell communication in sepsis livers in mice based on bulk RNA-seq. (**A**): Overall number of interactions over all 5 cell types in sham and CLP. (**B**): Overall interaction strength of interactions in sham and CLP, showing a clear increase in CLP. (**C**,**D**): CellChat analysis signaling heatmap (top 30) for outgoing signaling per cell type in sham (**C**) and in CLP (**D**), full figures in Figure S4. (**E**): CLP vs. sham per cell type signaling changes, circle/ring size is indicative of the number of interactions (incoming and outgoing) for the cell type. Conditions are represented by hollow rings for Sham and filled circles for CLP. The arrows indicated the direction of the change in interaction strength for the same cell type between the Sham and CLP conditions. (**F**): Top 30 pathways with the highest relative information flow in Sham (top 15) and in CLP (top 15). The top pathways show a relative information flow of one in either CLP or Sham, indicating that the active signaling is relevant in that condition only and is lost/absent in the other. For the full figure, see Supplemental Figure S5.

We used CellChat to obtain a global overview of communication interactions, predict interaction strengths in sham and CLP conditions, and perform top-level differential analyses. To retain only the most reliable results and taking into account that the CellChat method is optimized for single-cell data and was applied here using each biological replicate as a “cell”, we filtered on interactions that were supported by all three replicates. Overall, sepsis was found to increase both the total amount of interactions from 2030 to 4313 (Figure 2A) and the strength of those interactions by 2.2-fold (Figure 2B). This indicates that CLP is predicted to induce greater information flow between the five cell types and that the messages are more potent. Looking per cell type, we see that the primary driver of these changes is predicted to be the HSCs. In sham, CellChat finds that these HSCs play no signaling role (Figure 2C,E and Figure S4A), but after CLP treatment, the HSCs become a primary signaling hub, showing the most incoming and outgoing interactions of all cell types (Figure 2D,E and Figure S4B). We further investigated global, cell-type-independent, significant differences in signaling. This was visualized as the relative information flow (top 20 significant per condition shown in Figure 2F; corresponding unfiltered absolute information flow can be found in Figure S5). This approach identified ligands, receptors, and pathways, the activity of which differs most between conditions, revealing signals with a strong bias towards sham or CLP, as well as several pathways specific to one condition. Top signals specifically predicted to be active in the sham, but not in CLP, include steroid hormones (testosterone, DHT, androstenedione), as well as molecules involved in tissue homeostasis and ECM maintenance. We also find molecules involved in vascular maintenance (GP1BA) and barrier function and cell-cell contacts: OCLN, CLDN. In contrast, top signals specific to the CLP condition include molecules involved in ECM degradation and remodeling and tissue repair (TENASCIN, BMP10, PERIOSTIN, AGRN, MMP, MPZ), as well as apoptosis (TRAIL, TWEAK), and immune cell infiltration and activation (CD6, CX3C, and 12-oxo-LTB4). These molecules also support a strong HSC activation, as is known in CLP/sepsis: TENASCIN, PERIOSTIN, MMPs, and TWEAK are strongly associated with activated HSCs [39,40,41], while AGRN is more weakly but still clearly associated with activated HSCs. Taken together, these results suggest that sham livers rely primarily on hormonal and metabolic signaling, whereas CLP livers transition to communication dominated by stress responses, inflammation and tissue remodeling.

### 3.3. Tissue Remodeling and HSCs

**Figure 3:**
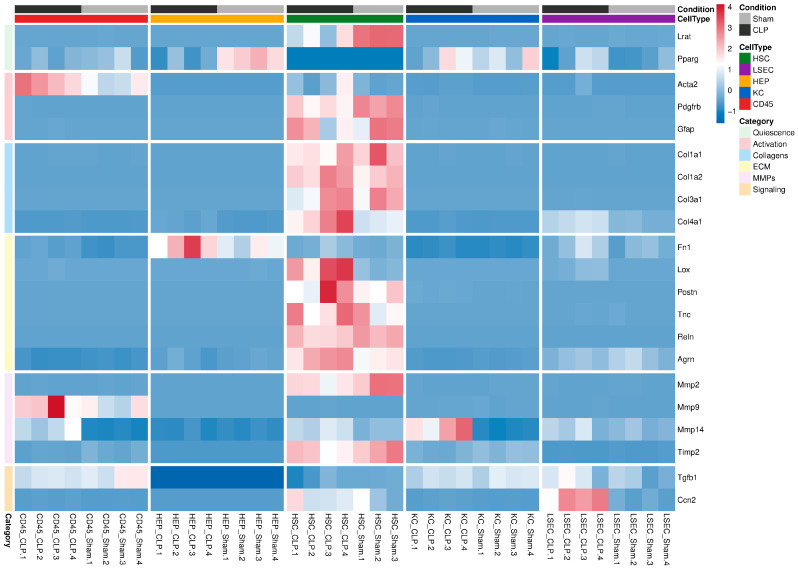
Heatmap of select genes for HSC status.

Based on differential expression results and a global overview from CellChat, HSCs emerge as a central predicted cell type in the hepatic CLP response. Now, we zoom in on this cell type and the cell-cell communications it participates in. We selected several genes indicative of HSC status, activity and functions (Figure 3). Overall, we see that the quiescence marker *Lrat* is significantly reduced on CLP. There is detectable expression of activation markers, both in Sham and CLP, which is likely related to the surgery itself. The expression of the classical activation marker gene *Acta2* (Alpha-smooth muscle actin) is detected, and slightly, but not significantly higher in CLP at this timepoint. The expression of various collagens is also seen on HSCs and altered, with some lower and others higher expressed after CLP, especially *Col4a1*, which shows an increase. The HSC also shows clear changes in the expression of several ECM proteins and related remodelers. Interestingly, the HEPs themselves show increased expression of fibronectin 1 (*Fn1*). The matrix metalloproteinase (*Mmp*) gene family was also selected for its role in ECM remodeling; however, among these, only *Mmp14* shows a slight trend towards increased expression in HSCs, while the expression of *Mmp2* and *Timp2* tends to decrease. Other cell types, such as KC and CD45, are the main contributors to *Mmp* gene expression. There are a few differences in the selected signaling molecules from HSC, but a strong increase in LSECs.

**Figure 4:**
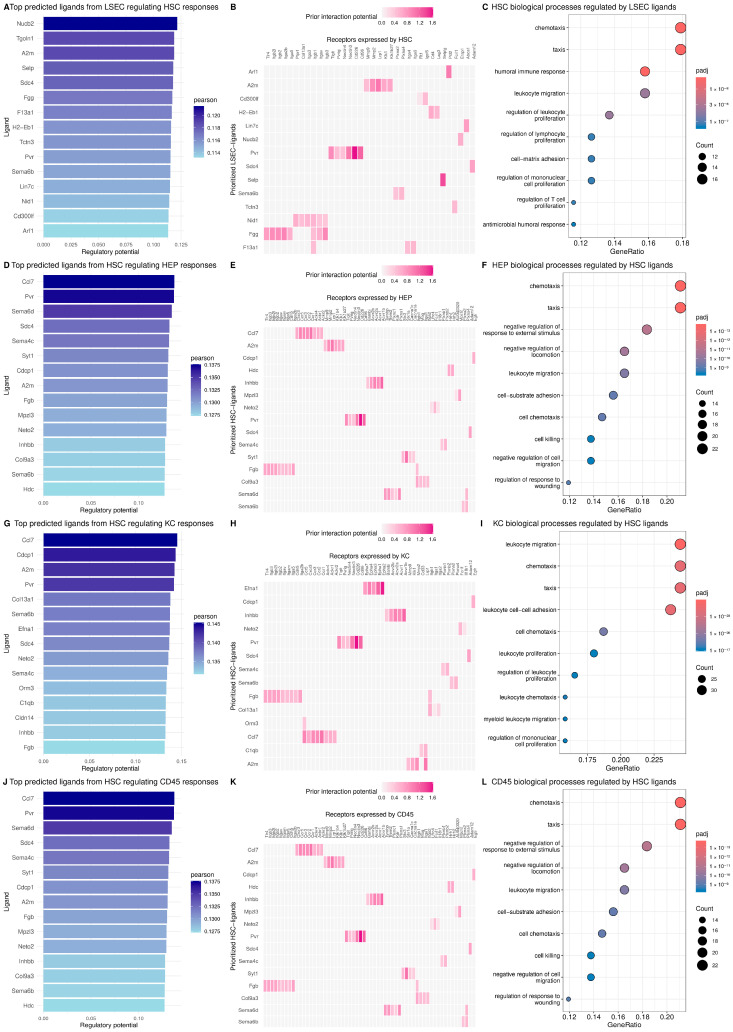
NicheNet predictions of cell-cell pairwise communications from the RNA-seq dataset. Panels (**A**,**D**,**G**,**J**) show the top 15 prioritized ligands by Pearson correlation of the known downstream profiles in the NicheNet database and observed gene expression profiles in the dataset, (**A**): top 15 prioritized ligands for LSEC → HSC, (**D**): top 15 prioritized ligands for HSC → HEP, (**G**): top 15 prioritized ligands for HSC → KC, (**J**): top 15 prioritized ligands for HSC → CD45, Top predicted ligands in LSEC-to-HSC. (**B**,**E**,**H**,**K**): prior interaction potential of ligands to receptors in the NicheNet database, filtered by our gene expression, showing which receptors and linked downstream targets are responsible for ligand signal in LSEC → HSC (**B**), HSC → HEP (**E**), HSC → KC (**H**), HSC → CD45 (**K**). (**C**,**F**,**I**,**L**): Functional (GO) enrichment results for receiver target genes regulated by the top 15 prioritized sender ligands for LSEC → HSC (**C**), HSC → HEP (**F**), HSC → KC (**I**), HSC → CD45 (**L**). padj: FDR adjusted *p*-value, Count: circle size, number of genes matched to the term, GeneRatio: ratio of number of genes annotated to the term relative to the input set (1 = 100%, 0.5 = 50%, 0 = 0%).

Based on the fact that we observe that direct signaling molecules are primarily expressed by LSECs, and considering that LSECs line form blood vessels and are thus the cell type that has first contact with any and all external stimuli, these observations suggest a hierarchical signaling cascade, with the LSECs as the initiating cell type with HSC as the central communication hub, and used NicheNet to further look into the LSEC → HSC and the HSC → other cells signaling pathways. NicheNet predicted the gene Nucleobindin-2 (*Nucb2*) as the ligand with the highest overall regulatory potential, indicating that it may be a primary driver of the differential gene expression in HSCs in CLP (Figure 4A). *Nucb2* plays a role in calcium signaling and is processed into the peptide nesfatin-1, which is involved in the regulation of food intake and energy homeostasis in the hypothalamus. Its role in HSCs is not well characterized, but it has been found to be an important player in liver dysfunction and liver disease, as reported by Yirui He et al. [42]. Based on the ligand-receptor heatmap (Figure 4B), nesfatin-1 seems to signal through, or use, Endoplasmic Reticulum Aminopeptidase 1 (*Erap1*) (Figure S8B) in some way. Analysis of the regulatory potential per target gene revealed only very minor differences between ligands, with overall low potential, suggesting functional redundancy among the ligands and receptors. This is visible on both the ligand-receptor heatmap (Figure 4B), where some ligands interact with many receptors and some receptors with many ligands, and from the ligand-target heatmaps (Figure S7A), showing that most ligands have a very low effect on almost all described target genes in HSCs, with the exception of Interleukin-1 beta (*Il1b*), which was predicted to be a strong target of Syndecan-4 (*Sdc4*) (Figure S7A). In general, HSC target genes predicted to be regulated by LSEC ligands are involved in inflammatory responses and positive regulation of the response to wound healing, indicating HSC activation (Figure 4C). Interestingly, in the context of LSEC-to-HSC signaling, several of these genes (such as *Sema6b*, *Sdc4*) and some of the functional enrichments (taxis, chemotaxis and matrix interactions) may also play a role in angiogenesis, consistent with the previous prediction of increased signaling between HSCs and LSECs in CLP (Figure 2E).

To further explore this cellular talk, we also analyzed the reverse direction (HSC-to-LSEC) using NicheNet and identified C-C motif chemokine ligand 7 (*Ccl7*) and Poliovirus Receptor (*Pvr*) as the top ligands activated by CLP in HSCs with impact on LSECs (Figure S6A). The NicheNet database shows that (*Ccl7*), predicted to signal through the *Ccr* and *Ackr* families of receptors (Figure S6B), has high regulatory potential for *Il1b*, Platelet-Derived Growth Factor C (*Pdgfci* and colony-stimulating factor 3 (*Csf3*), the latter of which is upregulated in LSECs and all other cells in CLP (Figures S6B,C and S8C,D). *Pdgfc* is a growth factor that acts as a mitogen and chemoattractant for mesenchymal cells, including HSCs, promoting their proliferation and activation and thereby contributing to fibrogenesis. Furthermore, a GSEA on LSEC target genes regulated by HSC ligands shows that they are largely associated with cell adhesion and cell migration (Figure S6D). Taken together, the first main prediction is that LSECs and HSCs communicate bidirectionally in CLP, contributing to HSC activation and enhanced angiogenesis, with *Nucb2* and *Ccl7* being potential mediators of this crosstalk.

We then investigated all other cellular crosstalk involving HSCs as the sender cell: HSC-to-HEP, HSC-to-KC and HSC-to-CD45 pairs. For HEPs as target cells, NicheNet predicted *Ccl7* (Figure S8E), *Pvr* (Figure S8F) and *Sema6d* (trough *Pexna1*) as the top ligands, with *Ccl7* showing signaling through the *Ccr* and *Ackr* family of receptors (Figure 4D,E) while showing high regulatory potential for *Adm* and *Csf3* targets (Figure S7B). *Adm* is upregulated by HEPs in CLP and may promote vasodilation by reducing the contractibility of HSCs, again suggesting a reciprocal interaction between HEPs and HSCs. Analysis of DEGs in HEP associated with HSC ligands revealed pathways related to inflammation and cell migration (Figure 4F).

For KCs as target cells, *Ccl7*, CUB Domain Containing Protein 1 (*Cdcp1*), Alpha-2 macrogloulin (*A2m*) and *Pvr* emerged again as top predicted ligands, involving the same receptors as in HEPs, with high regulatory potential for Periostin (*Postn*), *Il10*, *Csf3* and *Pdgfc* (Figure 4G,H and Figure S7C). The upregulation of *Pdgfc* and *Postn*, both resulting from HSC activation, again suggests a reciprocal interaction between KCs and HSCs. Pathway analysis of the DEGs related to HSCs further revealed processes associated with cell migration, consistent with our previous observation in Figure S2G (Figure 4I). Interestingly, in CLP, NicheNet predicts that HSCs communicate with all liver resident cell types via *Ccl7*, leading to the upregulation of *Csf3*. The latter is a protein that promotes tissue repair and neutrophil recruitment, and in the context of sepsis, its levels correlate with the severity and with sepsis-associated encephalopathy.

Finally, we investigated the signaling from HSC towards CD45 cells. The top ligand is *Ccl7*, followed by *Pvr* and *Sema6d*. Target regulation of top ligands is also diffuse and signals through a variety of receptors (Figure 4J–L and Figure S5D). The functions of target genes are involved in antimicrobial responses and cell migration.

In conclusion, we propose that HSCs may act as central regulators of the hepatic microenvironment in CLP-induced polymicrobial sepsis, orchestrating both inflammatory and regenerative processes through reciprocal interactions with HEPs, LSECs, KCs and CD45^+^ cells. Key candidate ligands mediating this predicted crosstalk include *Nucb2*, *Ccl7* and *Pvr*, with *Pdgfc*, Adrenomedulline (*Adm*) and *Postn* potentially contributing to further HSC activation. Furthermore, *Csf3* is upregulated by all liver cell types in response to HSC activation and may represent an interesting target for future investigations.

Integrating all of these analyses provides a hypothesis for a mechanistic link between intercellular signaling and specific cellular responses during sepsis. The strong pro-inflammatory signaling predicted in the CLP condition, in particular the interferons, directly corresponds to the upregulated interferon response pathway observed in HSCs. Furthermore, the identification of chemotactic ligands, such as *Cxc3*, *Ccl7* and members of the selectin gene family (e.g., *Selp*), could explain the involvement of the immune compartment and recruitment of circulating white blood cells. It is possibly responsible for the migratory and adhesive phenotype seen in KCs, while simultaneously recruiting new CD45. ECM remodeling is also a function for which several ligands are detected in CellChat (general MMP) and NicheNet, along with ligands affecting cell adhesion to other cells and to the ECM (semaphorins, selectins), as well as ECM remodeling, which supports ongoing remodeling of the liver in a dysregulated, fibrotic manner.

## 4. Discussion

Sepsis-induced liver dysfunction is a critical determinant of patient outcome, resulting from a complex and dysregulated inflammatory cascade involving multiple resident and infiltrating cells. This form of liver dysfunction arises from the normal liver’s response to clear inflammation, producing acute phase proteins and attempting to return to homeostasis. As the homeostatic processes are not restored, the liver cannot exit from this altered state and suffers from stress while failing to provide its normal metabolic functions. Currently, no specific therapeutics exist for the treatment of this form of liver dysfunction, which makes it a primary target of medical research [43,44]. In this work, we propose that this acute inflammatory and metabolically altered microenvironment can drive maladaptive cellular adaptations that, while attempting to mediate repair, may inadvertently prime the tissue for fibrogenic signaling and constrain long-term regenerative capacity.

While the general roles of different liver cells in inflammation are established, the specific microenvironmental cues that drive pathology remain poorly understood. In our study, differential expression analysis revealed a core set of 71 genes upregulated across all investigated cell types. This core set was enriched for pathways related to anti-apoptosis, responses to lipopolysaccharide (LPS), and reactive oxygen species (ROS) metabolism. Overall, HEPs show large transcriptional shifts, downregulating metabolic genes in favor of the acute phase response and other inflammatory genes. In particular, fatty acid metabolism was found to be strongly enriched in the downregulated set, a result driven by the significant downregulation of *Ppara* and *Pparg* transcription factors and their downstream targets. This failure of *Ppara* in sepsis conditions was described previously by Van Wyngene et al. [45]. We also show a radical reorganization of the hepatic intercellular communication network, with HSCs appearing as a key regulatory hub.

Despite making up only about 5–8% of the total liver cells, HSCs are known to exert a disproportionately large influence on liver biology, such as zonation, repair, metabolism and metabolic disorders, inflammation and fibrosis [11,46,47,48]. In sham conditions, HSCs are largely quiescent and uninvolved in cell-cell communication processes. However, in septic conditions, the communication network was predicted to shift drastically, with HSCs predicted to gain central receiver and sender roles in the tissue. In addition, the global inferred communication network showed increases in ECM and ECM remodeling proteins and pathways.

We propose a model with a complex interplay between environmental signals and LSECs, which are the first line of hepatic cells to sense systemic metabolic and inflammatory cues from portal blood circulation and are known to affect HSC status [48,49], producing signals leading to the observed transcriptional profile of the HSCs. Our NicheNet analysis identified *Nucb2*, which is strongly upregulated in LSEC (Figure S8A), as the top predicted LSEC-derived ligand involved in HSC responses. *Nucb2* encodes nucleobindin-2, the precursor to nesfatin-1, a peptide hormone increasingly recognized as a potent regulator of hepatic lipid and glucose metabolism. In addition, nesfatin-1 has been described to be involved in NAFLD, where it serves as a direct protective factor and is supposed to suppress HSC activation by blocking Transforming Growth Factor Beta 2 (*Tgfb2*) [49,50]. However, other signals are not inhibitory, and we also clearly see that HSCs are highly active in signaling.

The HSCs show a strong increase in translation machinery and may broadcast a fibrogenic and chemotactic signature to the rest of the liver. NicheNet predicted that HSCs dominate outgoing signaling via proinflammatory chemokines (e.g., *Ccl7*) and ECM regulators (e.g., *Col9a3*, *Sdc4*) directed at HEPs, KCs, and CD45. HSC ligands would predominantly drive pathways related to chemotaxis, leukocyte migration, and cell-matrix adhesion. Furthermore, the signaling from HSC-derived ECM regulators to HEPs is particularly noteworthy when considering avenues for further investigation. Consistent with the previous literature, altered ECM composition and stiffness directly influence HEP behavior, driving their dedifferentiation and further constraining their metabolic and regenerative properties [50,51].

The added value of our work lies in combining sorted bulk profiling with multi-tool communication inference, uncovering HSC as a mediator amplifying immune-HEP crosstalk, which was absent in prior single-cell RNA-seq studies focused on individual cell states. This approach offers predictions of druggable targets for sepsis-induced liver abnormalities. By identifying the LSEC → HSC (*Nucb2*) and HSC → Tissue (*Ccl7*) axes, we highlight specific nodes where this maladaptive signaling could be altered or blocked.

Importantly, the early fibrogenic priming and HSC activation we observe in acute sepsis show remarkable mechanistic similarity to chronic conditions like NASH and liver fibrosis. Understanding sepsis not just as an acute inflammatory event, but as a trigger for a metabolically altered, fibrogenic microenvironment, opens new therapeutic avenues. A recent study demonstrated the feasibility of specifically targeting activated fibroblasts and HSCs using lipid nanoparticles in the context of fibrosis [52,53], which may be considered for other conditions like sepsis [54,55].

However, we do recognize that our approach has several limitations, which must be taken into account. Firstly, the findings are based on CLP-induced polymicrobial sepsis in mice, which is a widely used and clinically relevant model, but does not fully capture the complexity and heterogeneity of human sepsis. Therefore, extrapolation of these results to human disease should be done with caution due to species-specific differences in immune responses and disease progression. Secondly, transcriptomic profiling was performed using bulk RNA-seq of sorted cell populations, which provides averaged gene expression signals and does not resolve cellular heterogeneity at the single-cell level. In addition, there is no guarantee that observed transcriptional changes actually relate to protein abundances, but as the elevation of mRNA production almost always precedes increases in protein levels, we are confident that increases in gene expression correlate well with increases in ligand. Thirdly, there is the mixture of cells in the niche; a single ligand is very likely to be produced by more than one cell type. Considering the layout of the space of Disse, secreted ligands may diffuse to not immediate neighbors and might be a mix from multiple sources. An example of this is indeed the *Ccl7* gene, while upregulated in HSC by CLP, Figure S8E clearly illustrates that KC also strongly induces this gene and has higher total expression. Finally, there is the computational and predictive nature of our data. The inferred cell-cell communication networks are based on transcriptomic profiling and ligand–receptor databases, and therefore do not directly prove that these interactions occur or are functionally relevant in vivo. The proposed signaling axes should thus be interpreted as hypothesis-generating and will require future validation at the protein, spatial, and functional levels.

## 5. Conclusions

Overall, we provide a cell-type-resolved view of the hepatic response to polymicrobial sepsis. Our study highlights extensive inflammatory, metabolic, and intercellular communication remodeling occurring in the liver microenvironment after CLP. We identify a limited set of upregulated genes shared across all cell types as well as a set of related pathways showing anti-apoptotic, LPS-response, and oxidative stress-related signatures, together with a clear suppression of hepatocyte metabolic programs, particularly those related to fatty acid metabolism and PPAR signaling.

**Figure 5:**
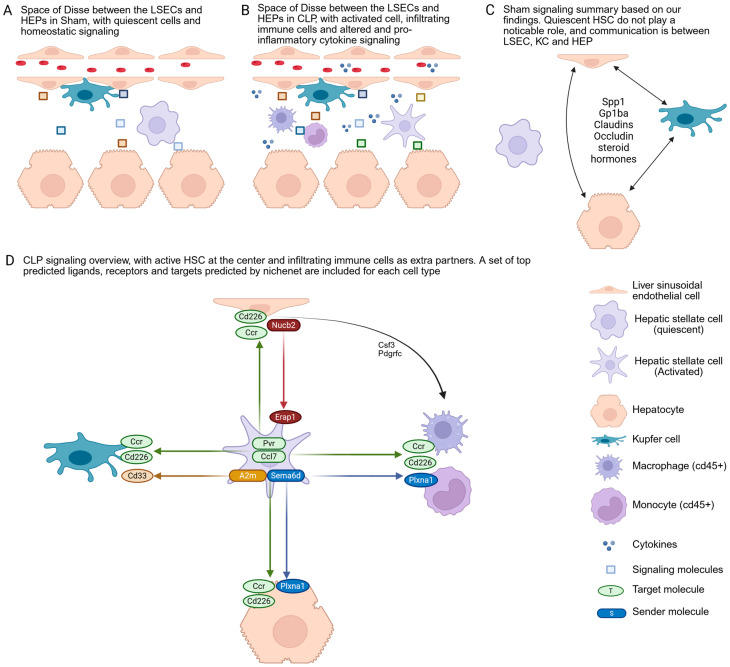
Schematic overview of cell-cell communication evolution. (**A**) Space of Disse in Sham conditions, with quiescent HSC and no inflammatory signaling. (**B**) Space of Disse in CLP conditions, with active HSC, infiltration of additional CD45 cells, and no inflammatory signaling. (**C**) Homeostatic signaling as found in Sham by CellChat. (**D**) CLP-induced signaling changes, with a selection of the primary signaling molecules shown. LSEC uses Nucb2 to signal to HSC. HSCs are activated and use Pvr and Ccl7 to signal all other cell types and A2m, and use Sema6d to signal more specifically. LSEC, in turn, expresses various chemoattractants for non-resident immune cells.

Cellchat and nichenet analyses also suggest a deep restructuring of cell-cell communication even at this early stage in sepsis. The liver microenvironment may acquire features of regeneration pathway activation and early fibrogenic priming. In particular, predicted LSEC-to-HSC and HSC-to-tissue signaling axes, including Nucb2- and Ccl7-associated pathways, may contribute to HSC activation, extrahepatic immune cell recruitment, ECM remodeling, and altered hepatocyte function. We propose a schematic summary with some of the predicted ligands, receptors and targets in Figure 5.

Although the computational nature limits the direct applicability of our findings and further validation in single-cell datasets, protein-level analyses, spatial models, and human sepsis samples will be required, this work identifies hepatic stellate cells and their communication networks as potential therapeutic entry points. Targeting maladaptive HSC-centered signaling may therefore represent a promising strategy to mitigate sepsis-induced liver dysfunction and improve restoration of hepatic homeostasis.

## Data Availability

RNA-seq data are available on the Gene Expression Omnibus (GEO) archive with the ID GSE311736.

## Supplementary Materials

The following supporting information can be downloaded at: https://www.mdpi.com/article/10.3390/cells15110968/s1, Figure S1: Supplemental RNA-seq data for group clustering and cell type purity. (**A**): Expression of HEP marker *Alb*. (**B**): Expression of LSEC marker *Stab2*. (**C**): Expression of HSC marker *Lrat*. (**D**): Expression of KC marker *Clec4f*. (**E**): Expression of cholangiocyte marker *Epcam*. (**F**): Expression of *Lbp*. (**G**): Expression of *Tnf*. (**H**): Expression of *Crp*. (**I**): Expression of *Acta2*. J: Upset plot of the overlap of differential expressed genes between cell types. Figure S2: Functional enrichment for up- and downregulated genes per cell type between CLP and SHAM (pt 1) (**A**): Functional enrichment (GO) top 10 pathways of genes upregulated in HEPs. (**B**): Functional enrichment (GO) top 10 pathways of genes downregulated in HEPs. (**C**): Functional enrichment (GO) top 10 pathways of genes upregulated in LSECs. (**D**): Functional enrichment (GO) top 10 pathways of genes downregulated in LSECs. (**E**): Functional enrichment (GO) top 10 pathways of genes upregulated in HSCs. (**F**): Functional enrichment (GO) top 10 pathways of genes downregulated in HSC. (**G**): Functional enrichment (GO) top 10 pathways of genes upregulated in KCs. (**H**): Functional enrichment (GO) top 10 pathways of genes downregulated in KCs. Figure S3: Functional enrichment for up- and downregulated genes per cell type between CLP and SHAM (pt 2) (**A**): Functional enrichment (GO) top 10 pathways of genes upregulated in CD45^+^ cells. (**B**): Functional enrichment (GO) top 10 pathways of genes downregulated in CD45^+^ cells. (**C**): Functional enrichment (GO) top 10 pathways of genes upregulated in all cell types. Figure S4: Full plots of the subset shown in Figure 2C,D. (**A**): Full plot of the CellChat analysis signaling heatmap for outgoing signaling per cell type in Sham. (**B**): Full plot of the CellChat analysis signaling heatmap for outgoing signaling per cell type in CLP. Figure S5: Full plot of the information flow from CellChat. Genes are ranked by relative information flow in the same order as shown if Figure 2F. Statistically significant condition bias was tested by the Wilcoxon test, significant results are colored in the same color as the more active condition, non-significant results are black text. Figure S6: Plots of ligand-target potential interactions showing direct single ligand target pearson correlation values. (**A**): Top ligand target interactions in LSEC-to-HSC. (**B**): Top ligand target interactions in HSC-to-HEP. (**C**): Top ligand target interactions in HSC-to-KC. (**D**): Top ligand target interactions in HSC-to-CD45^+^. Figure S7: NicheNet results for the HSC to LSEC sender/receiver interaction. (**A**): Top predicted ligands in HSC-to-LSEC. (**B**): Top ligand receptor interactions in HSC-to-LSEC. (**C**): Ligand-target regulatory potential in HSC-to-LSEC. (**D**): Functional enrichment for LSEC targets. Figure S8: Supplemental RNA-seq data for gene expression. (**A**): Expression of *Nucb2*. (**B**): Expression of *Erap1*. (**C**): Expression of *Pdgfc*. (**D**): Expression of *Csf3*. (**E**): Expression of *Ccl7*. (**F**): Expression of *Pvr*. (**G**): Expression of *Cd226*. H: Expression of *A2m*. I: Expression of *Plxna1*. Table S1: DESeq2 differential gene expression results for the HEP. Table S2: DESeq2 differential gene expression results for the LSEC. Table S3: DESeq2 differential gene expression results for the HSC. Table S4: DESeq2 differential gene expression results for the KC. Table S5: DESeq2 differential gene expression results for the CD45. Table S6: Lists used for function overrepresentation analyses, organized per column.
