# Supplementary figures and images for: Polymicrobial Sepsis-Induced Changes in Hepatic Stellate Cell Communication in Male C57BL/6J Mice
Source: Cells. 2026 May 24;15(11):968. doi: 10.3390/cells15110968 (PMC13256657; doi:10.3390/cells15110968)

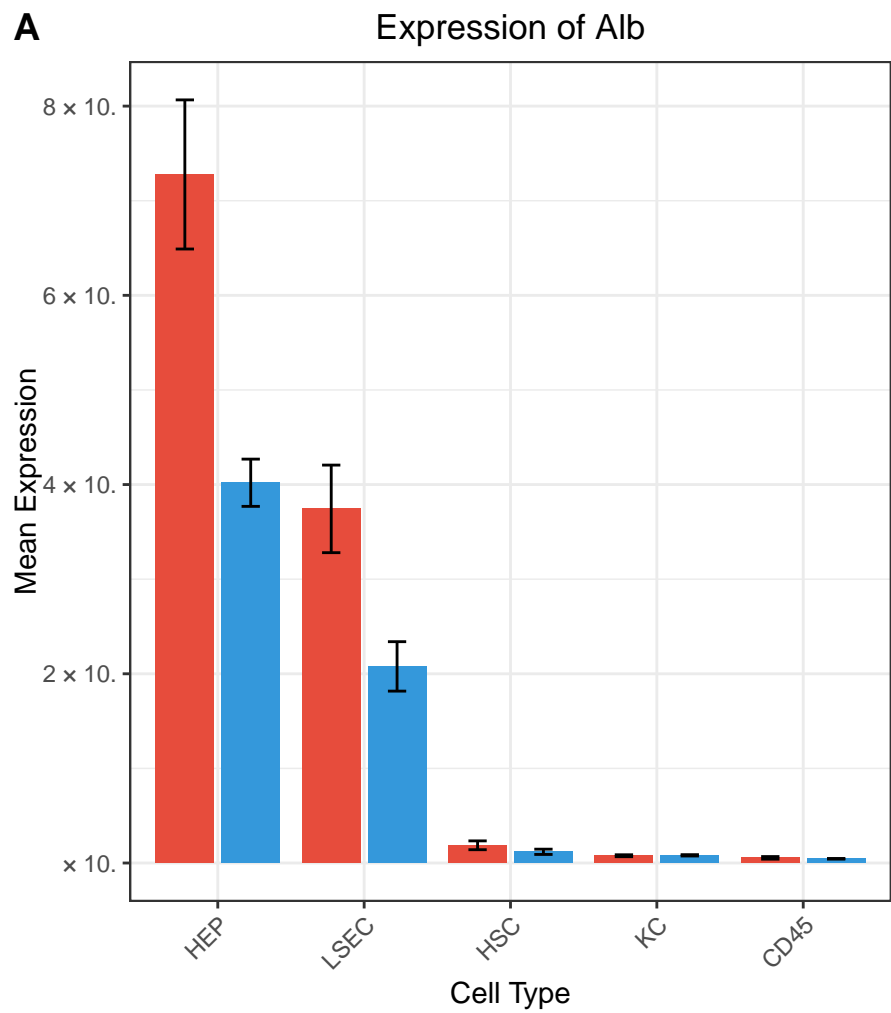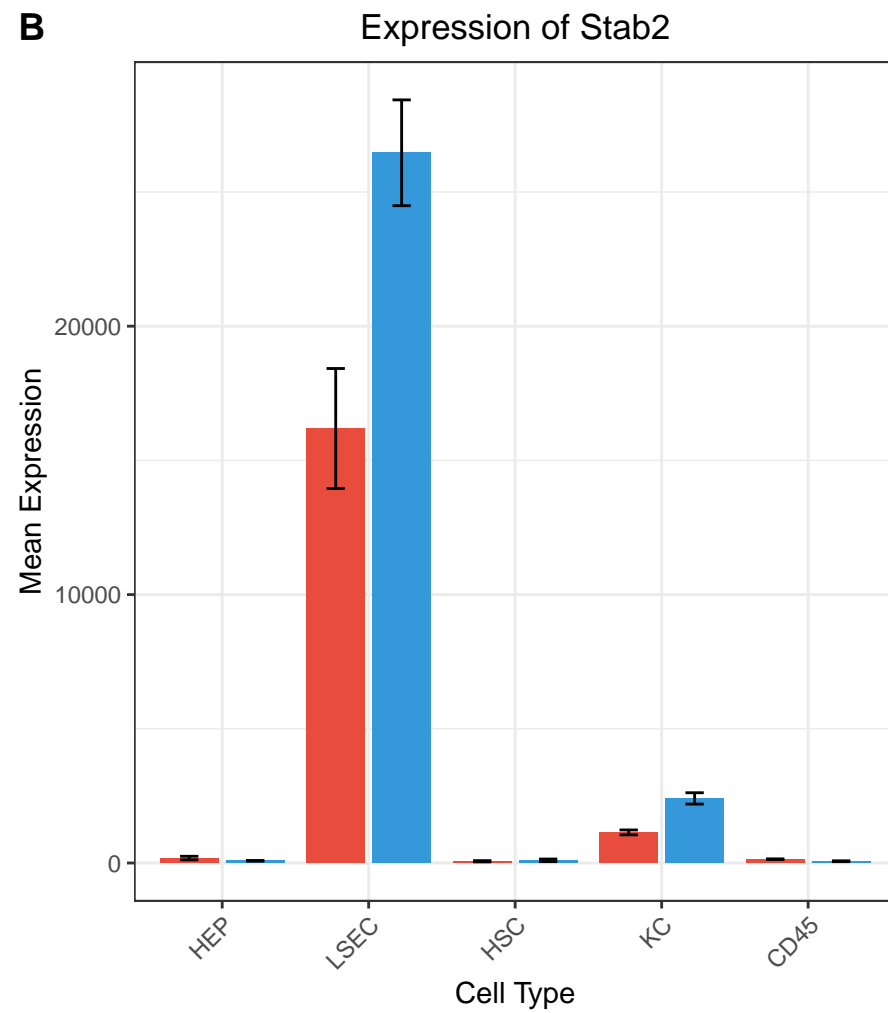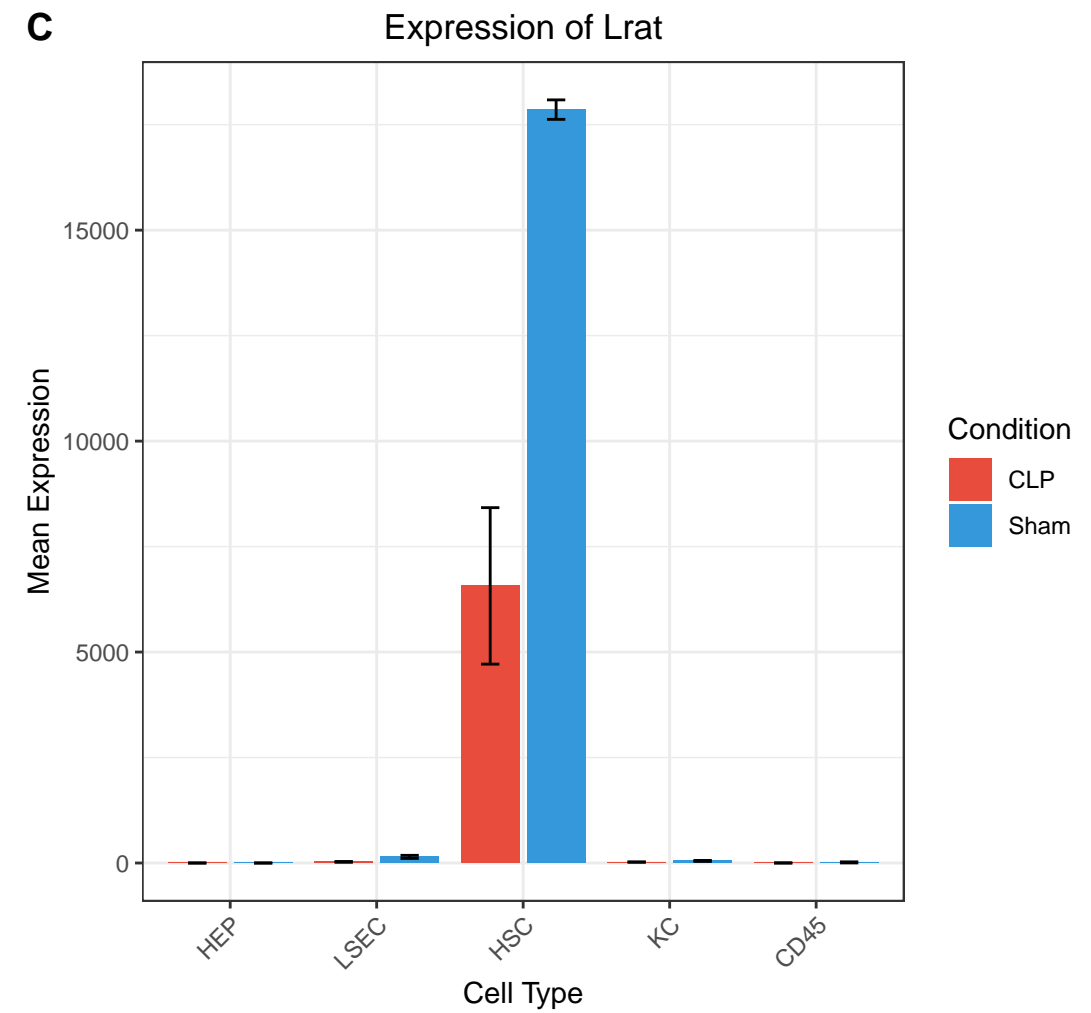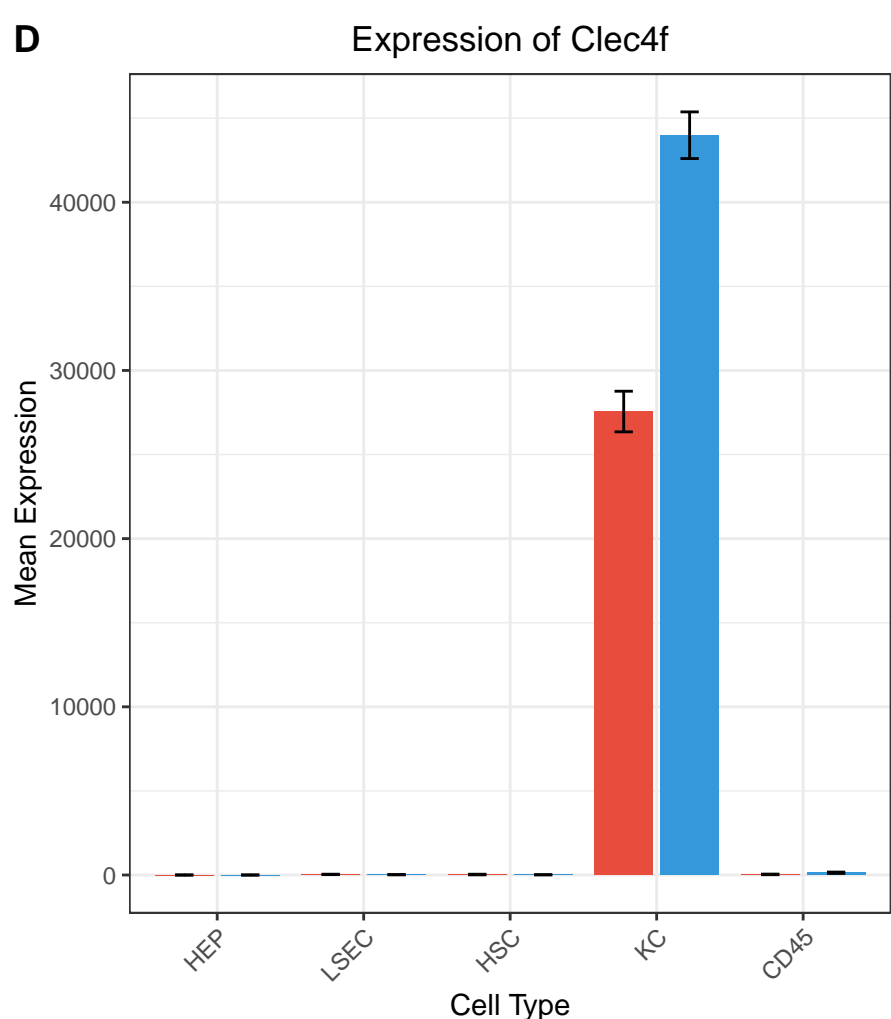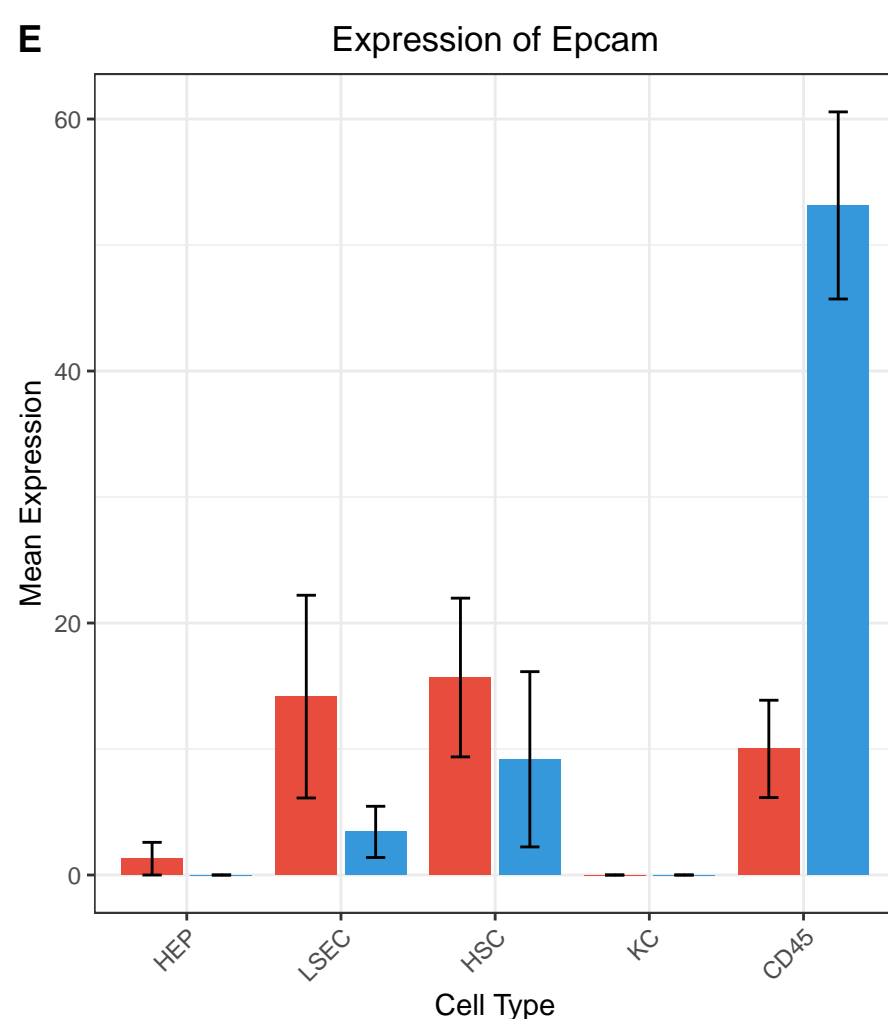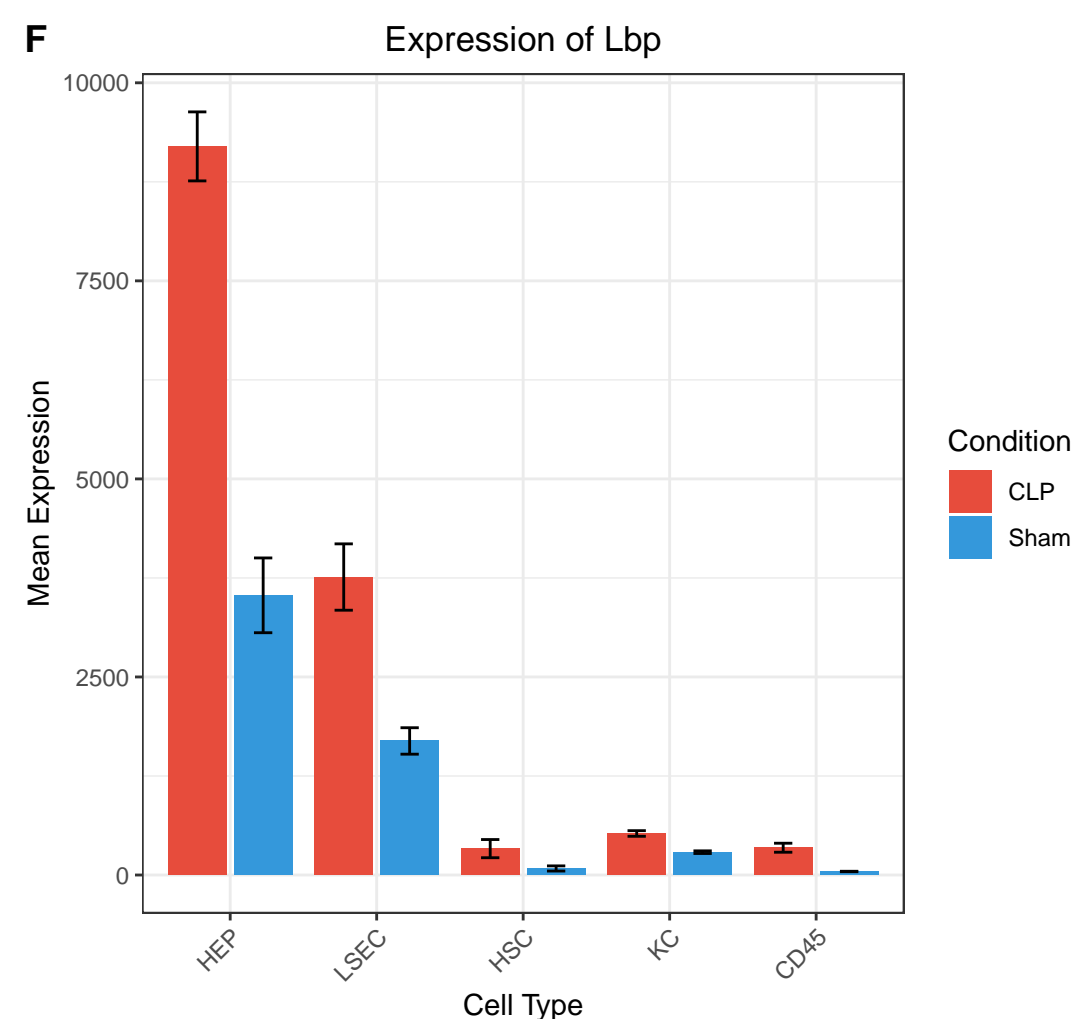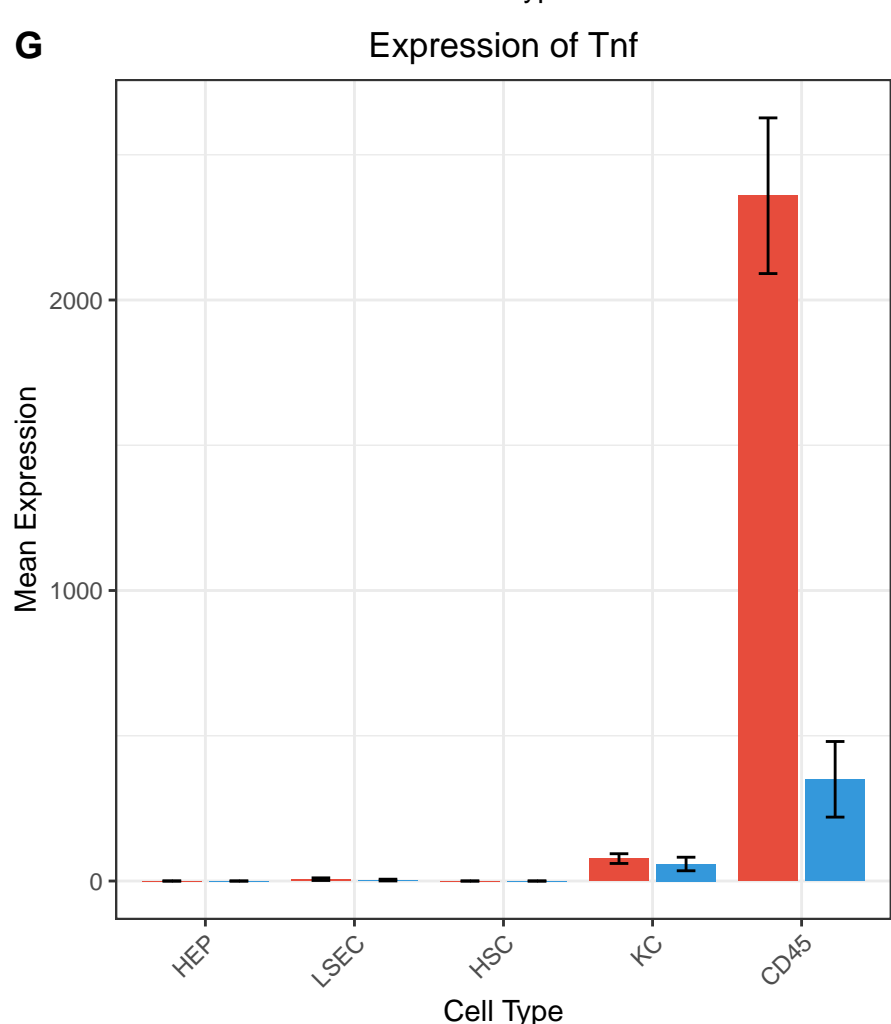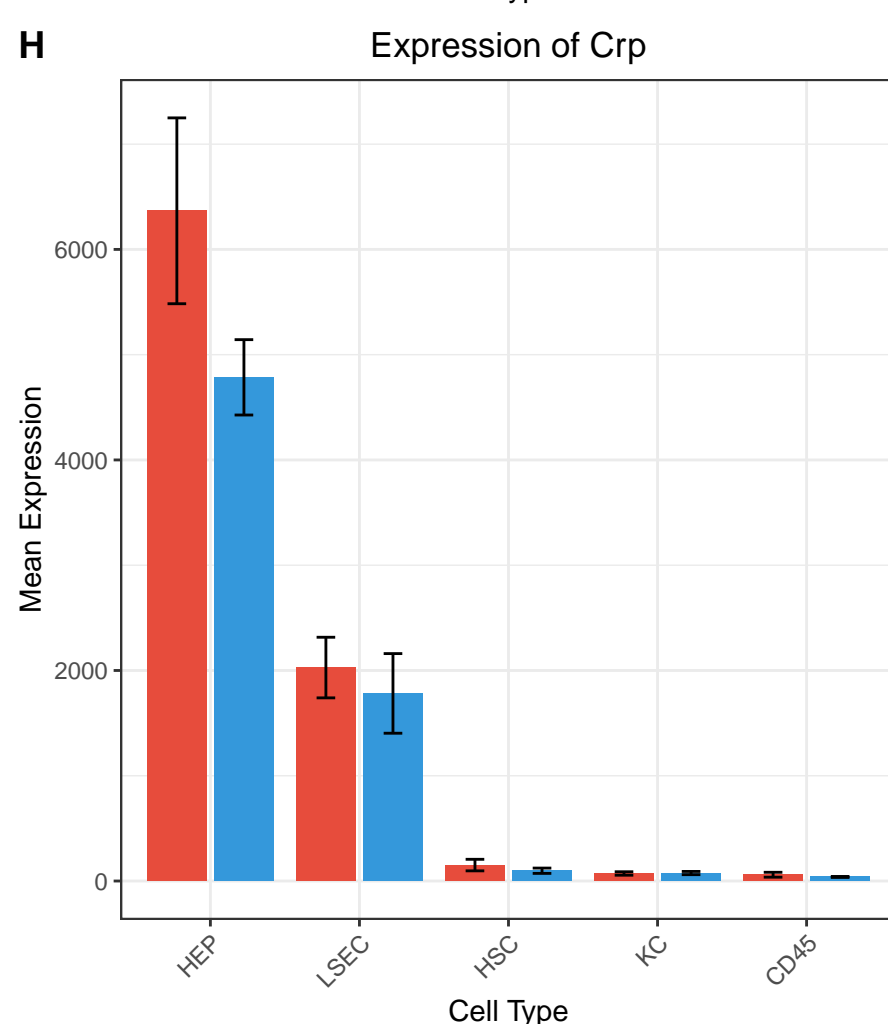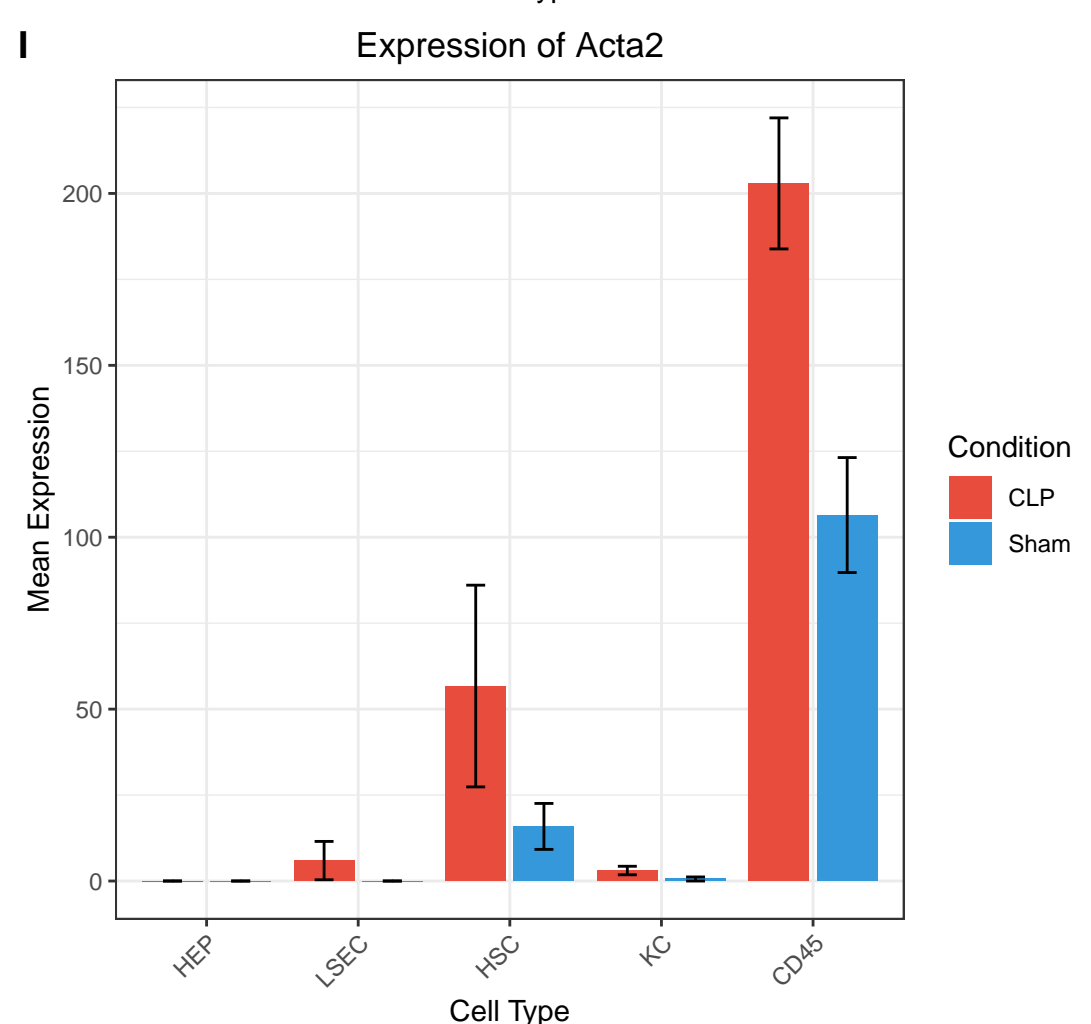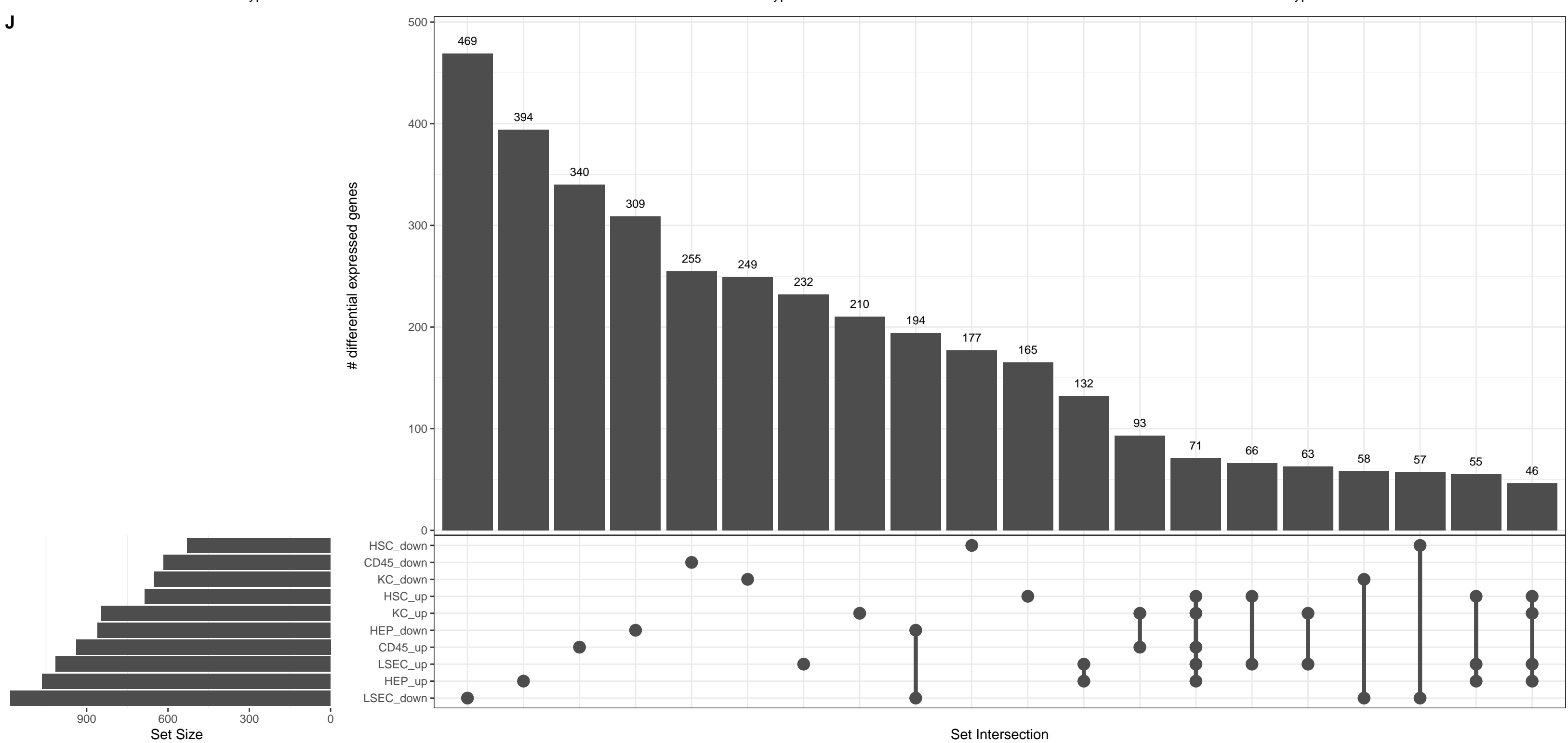

Supplement: Supplementary file 1 [file cells-15-00968-s001.zip › Figure_S1.pdf]

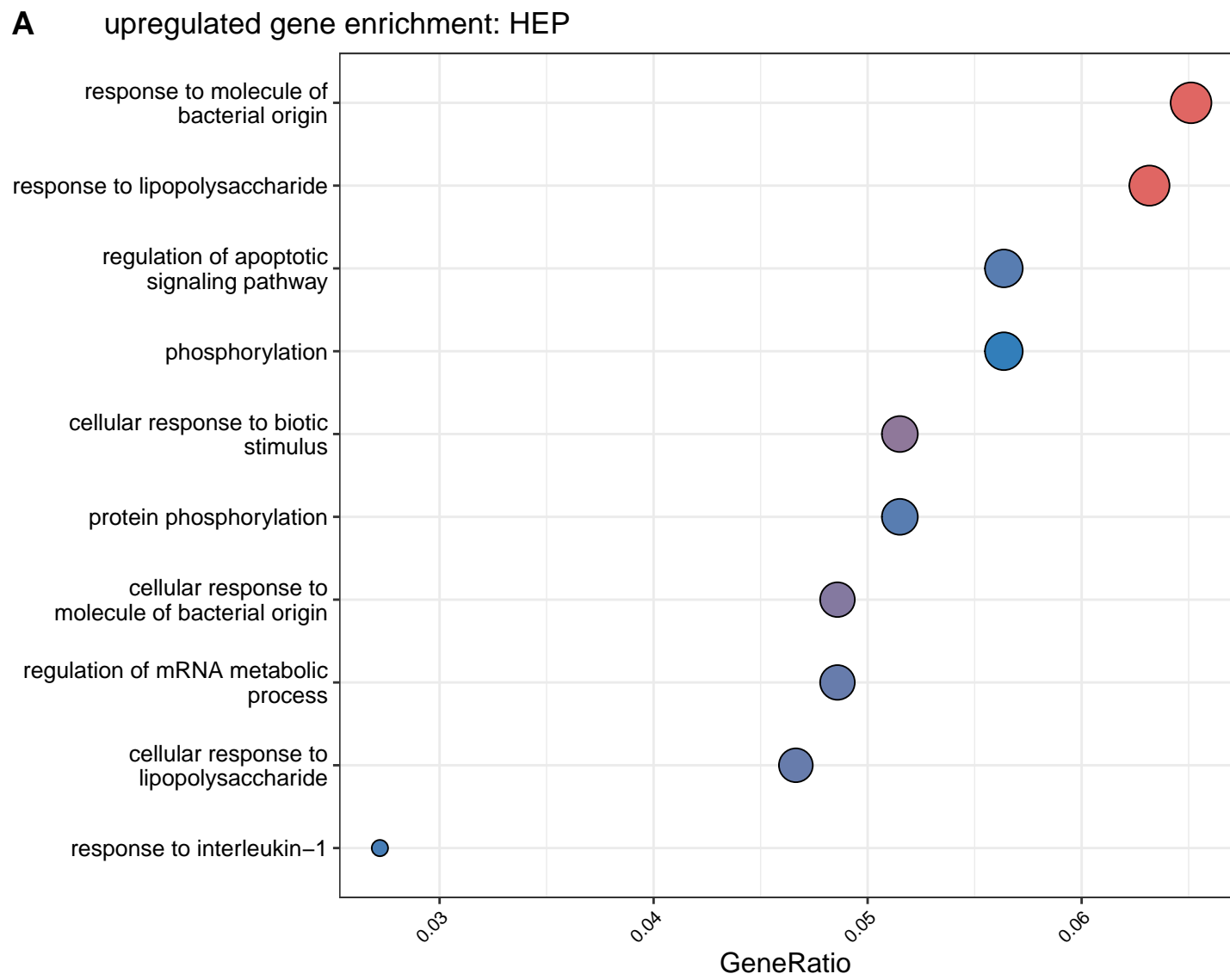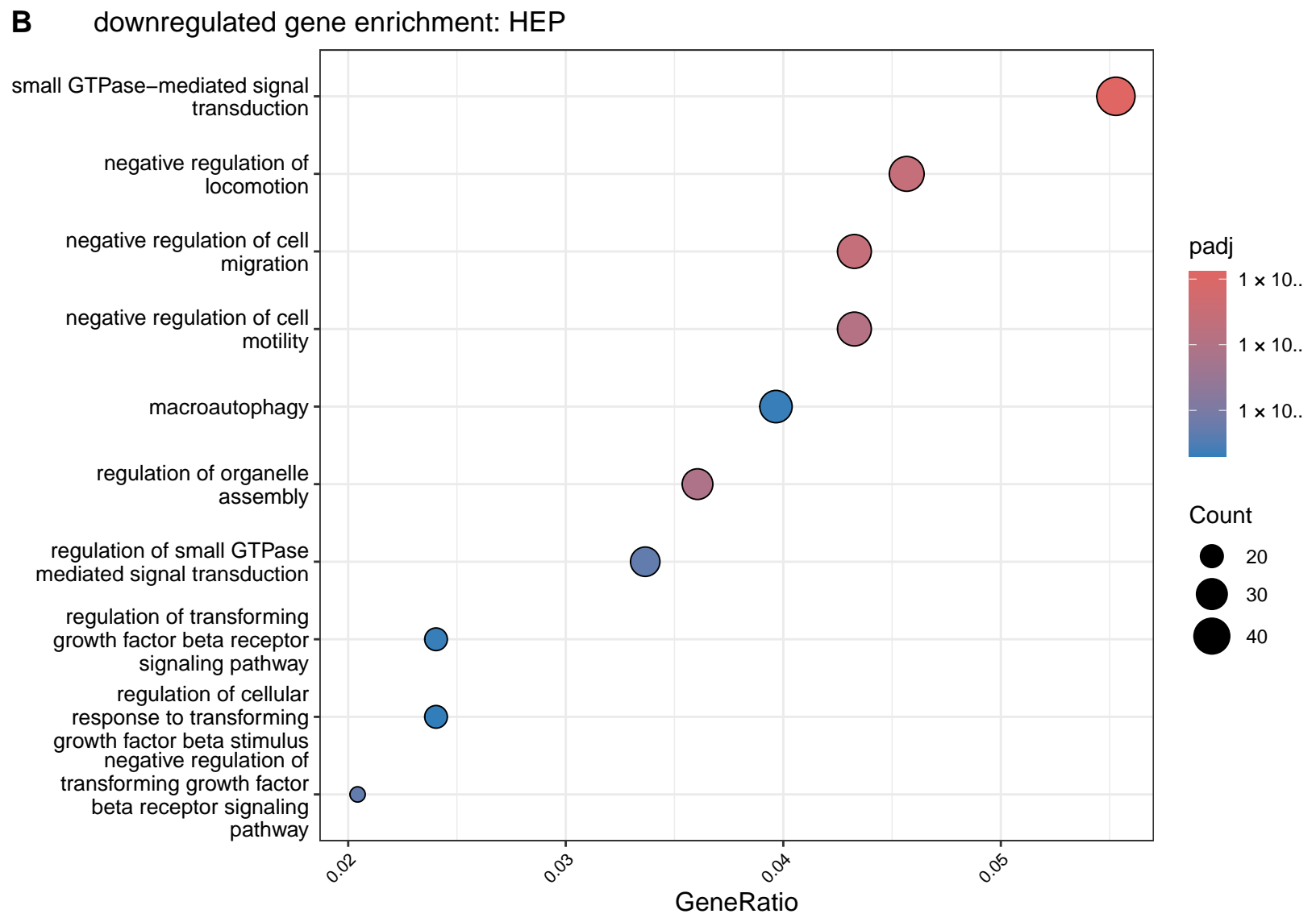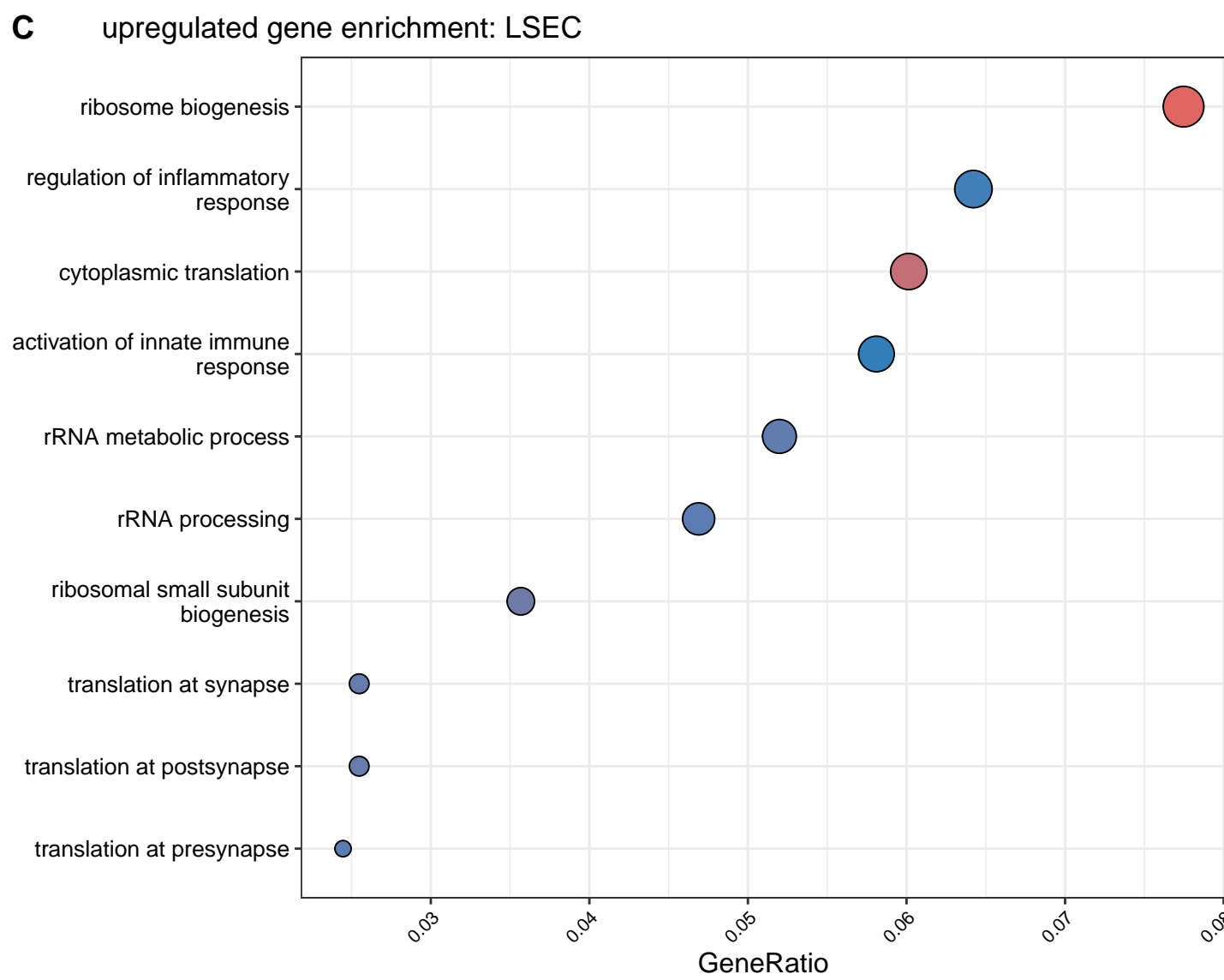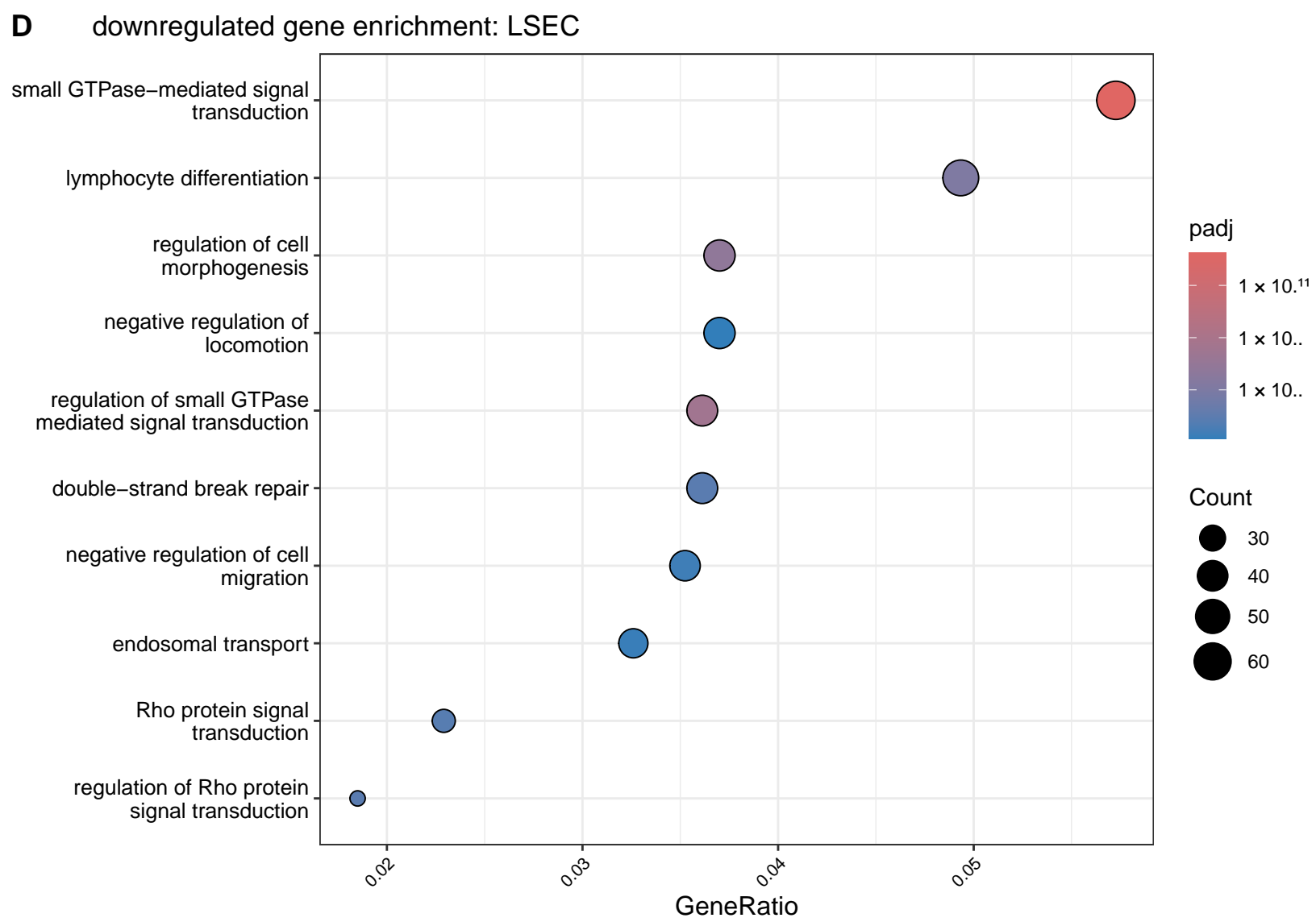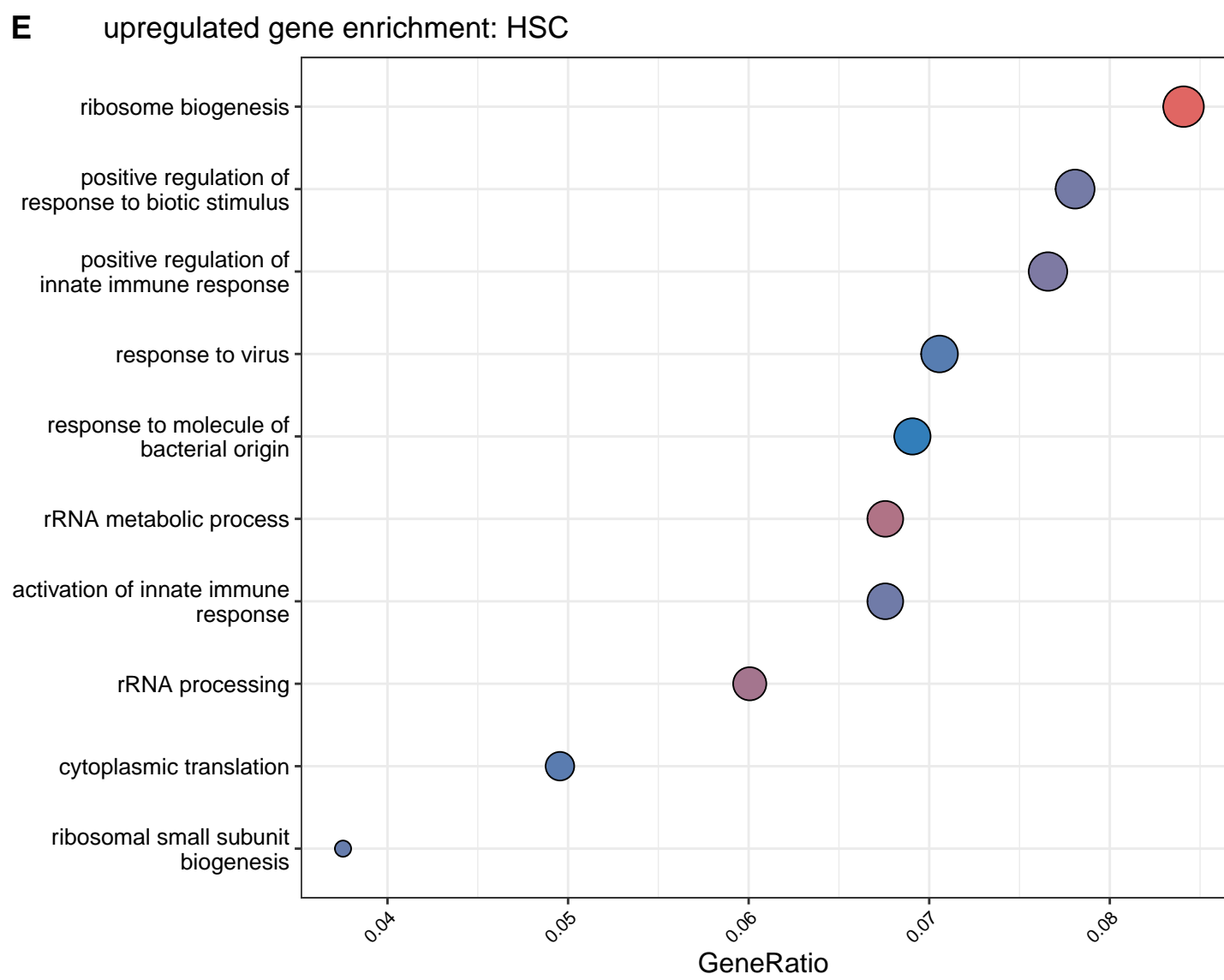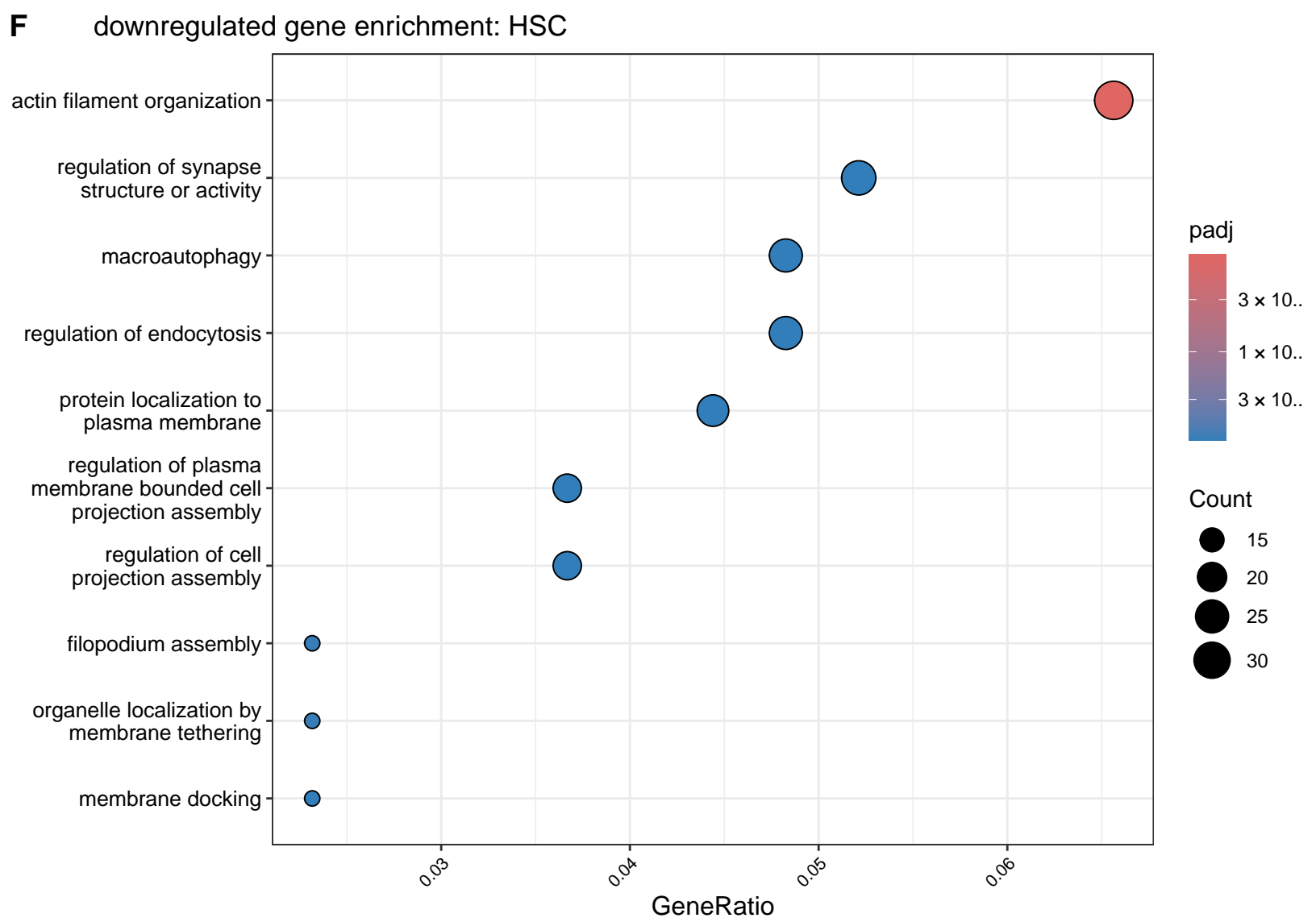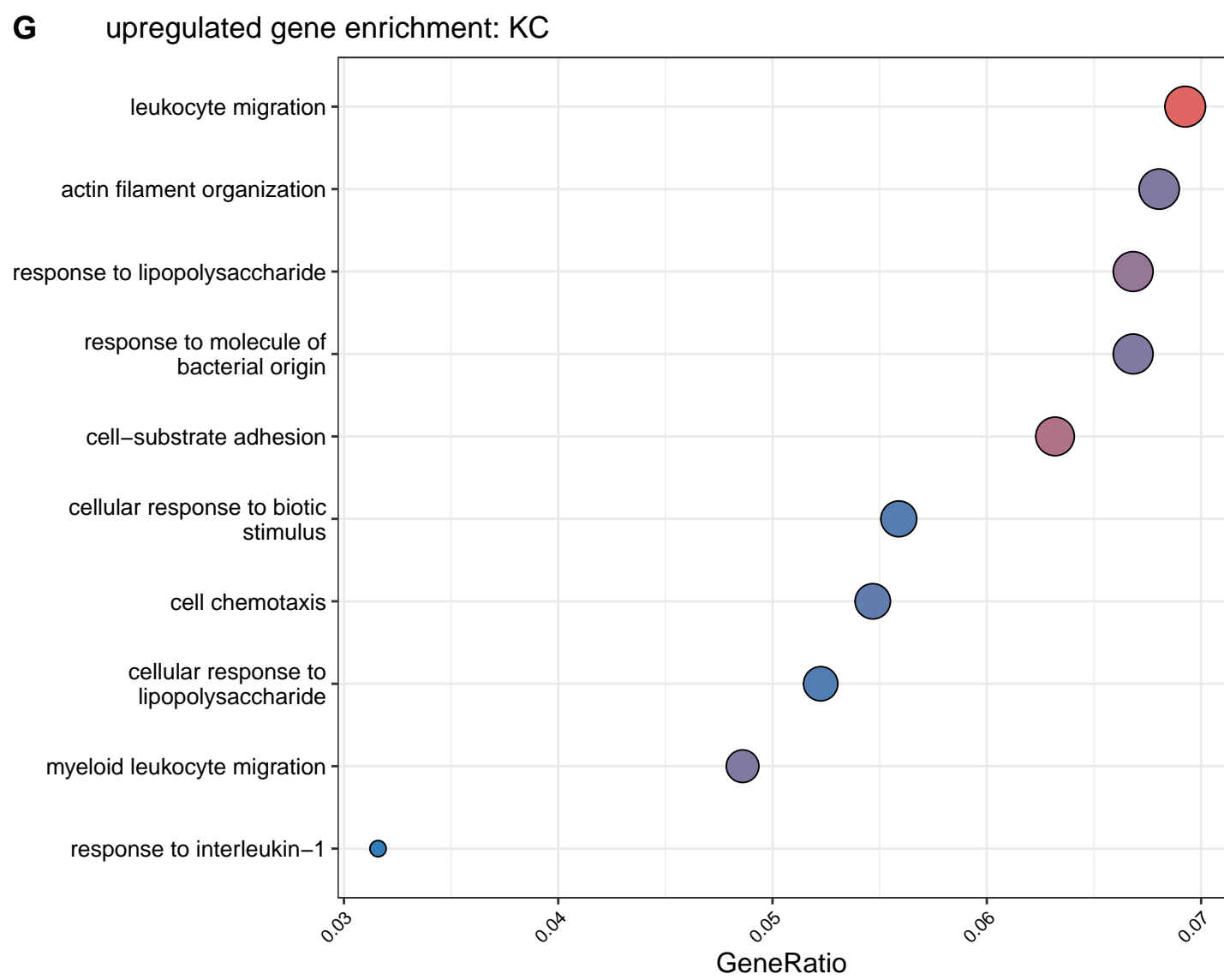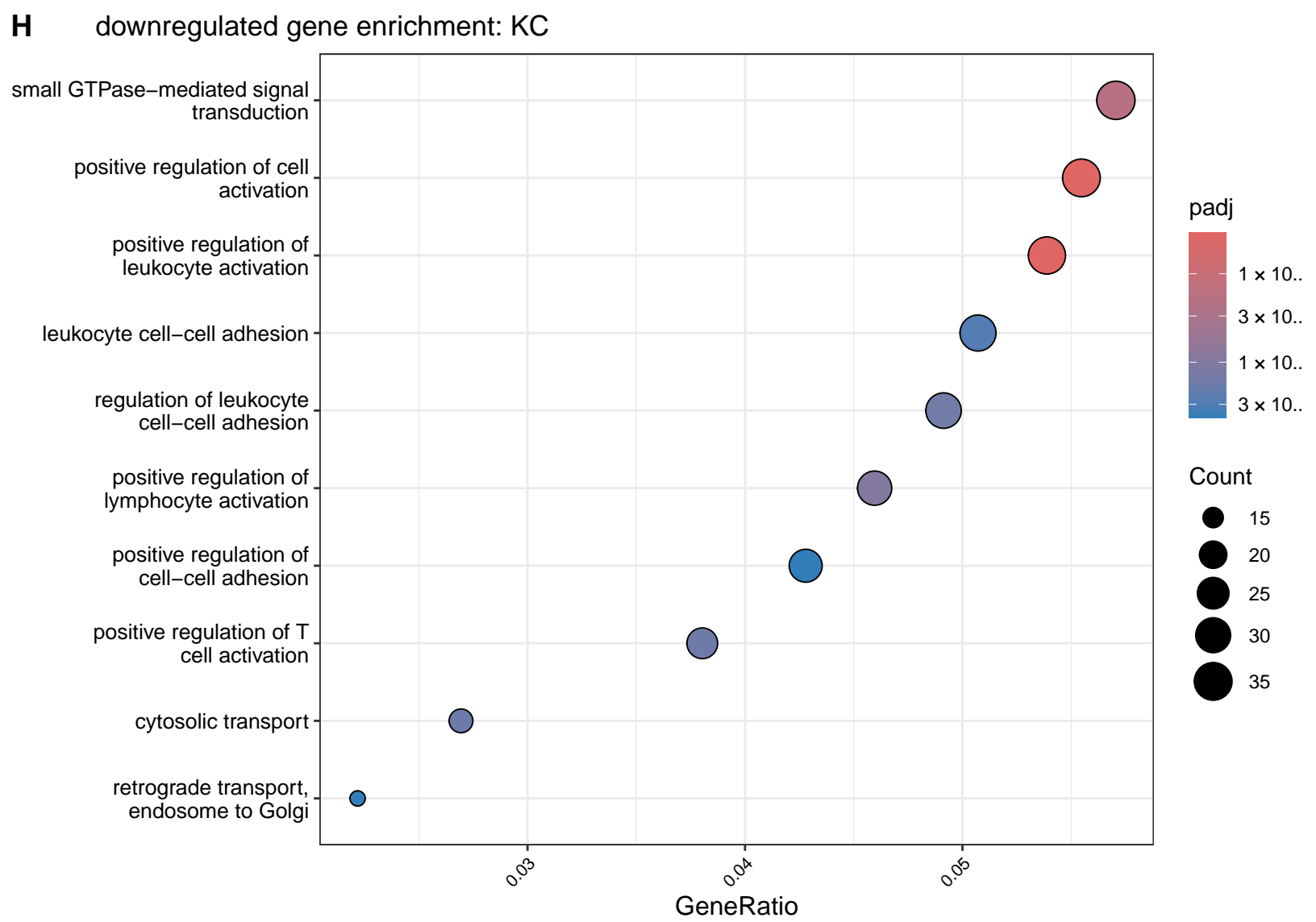

Supplement: Supplementary file 1 [file cells-15-00968-s001.zip › Figure_S2.pdf]

**A** upregulated gene enrichment: CD45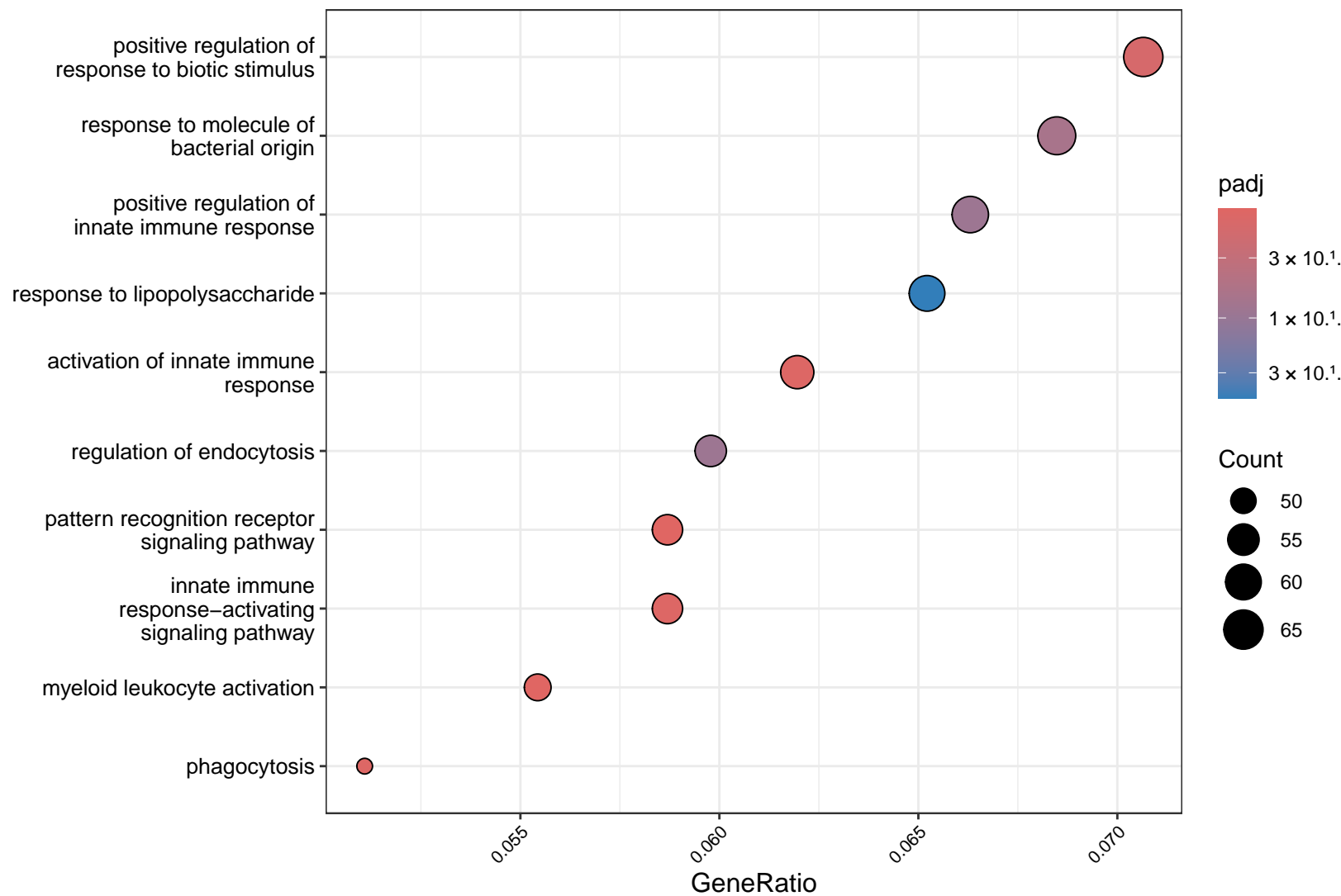**B** downregulated gene enrichment: CD45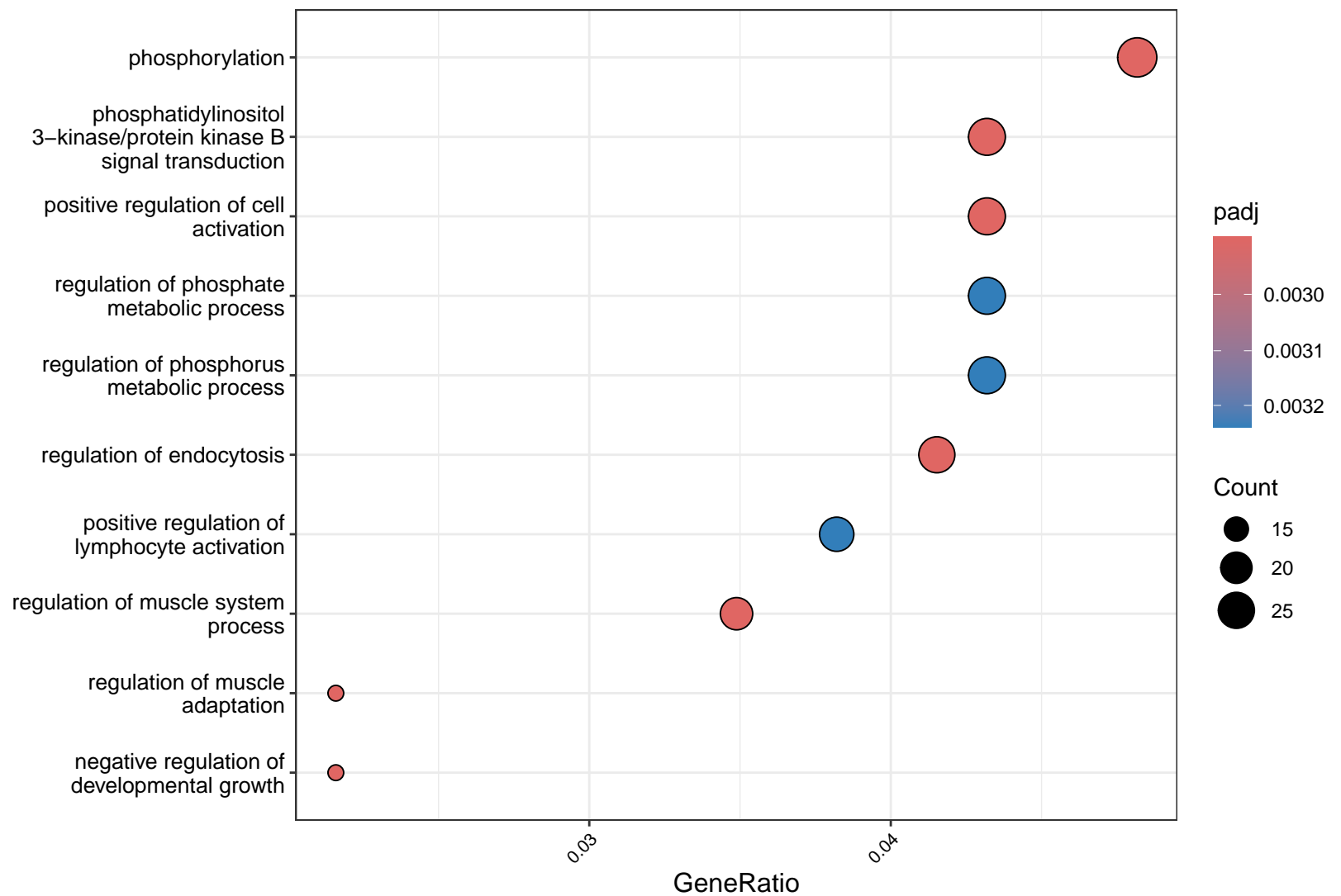

Supplement: Supplementary file 1 [file cells-15-00968-s001.zip › Figure_S3.pdf]

**A**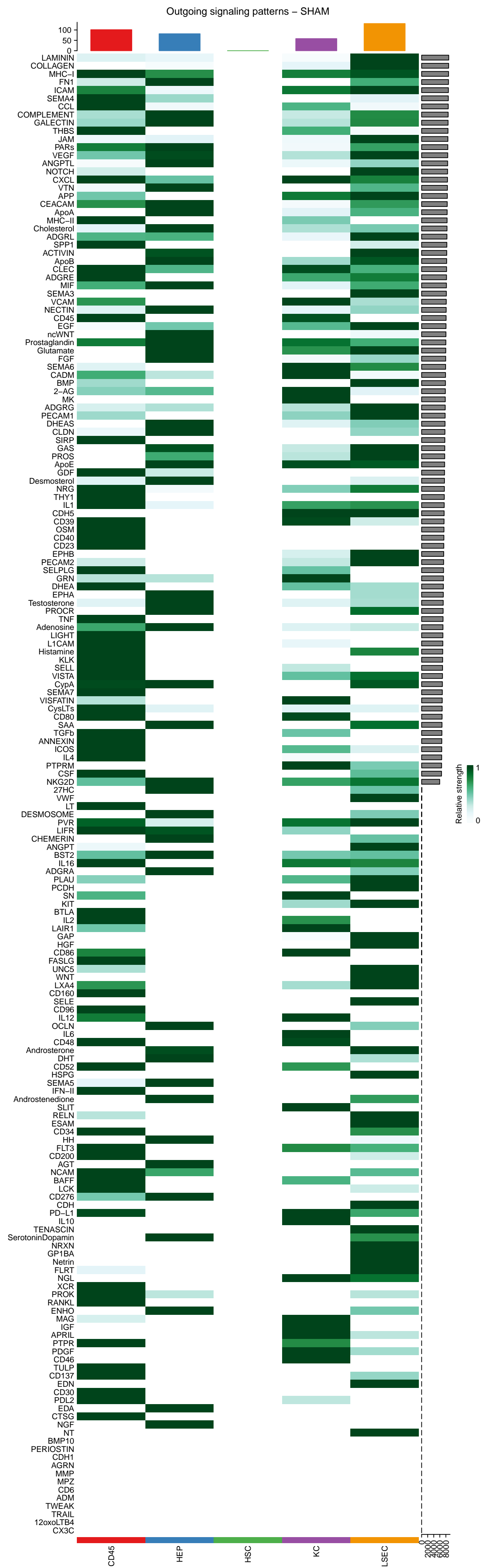**B**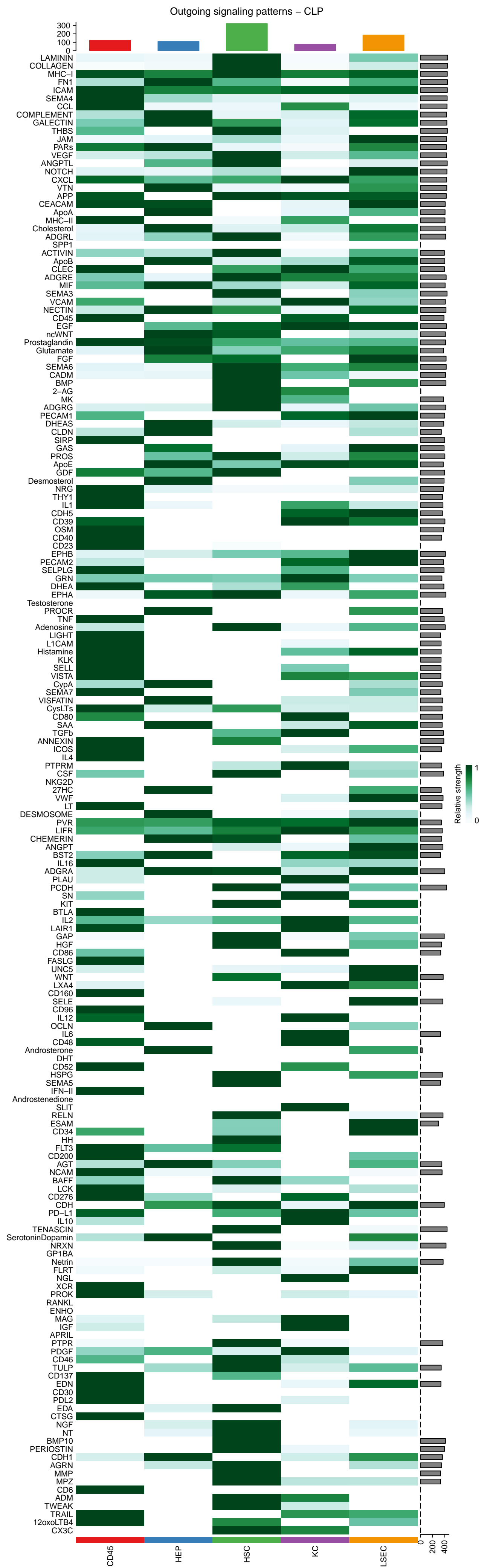

Supplement: Supplementary file 1 [file cells-15-00968-s001.zip › Figure_S4.pdf]

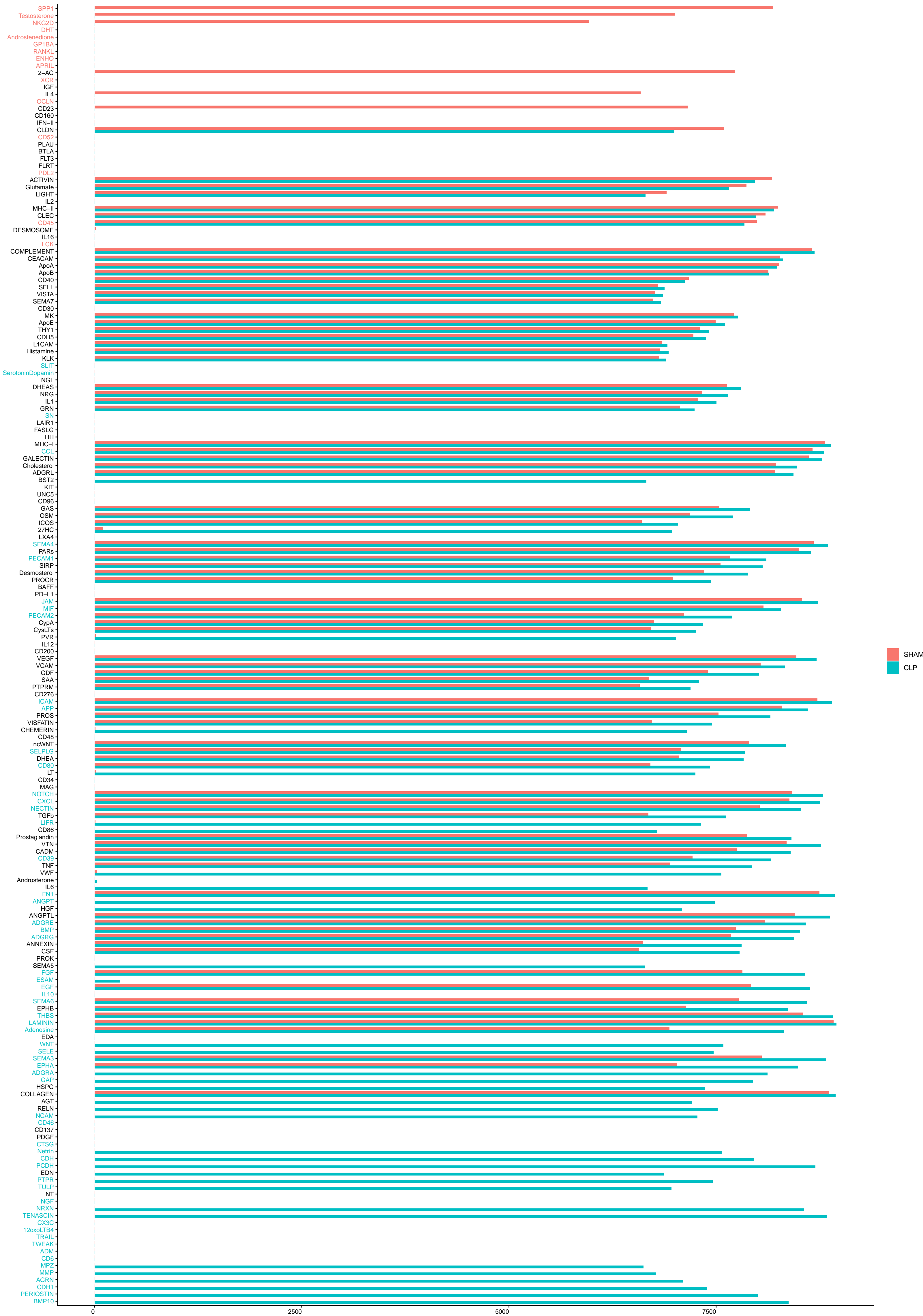

Supplement: Supplementary file 1 [file cells-15-00968-s001.zip › Figure_S5.pdf]

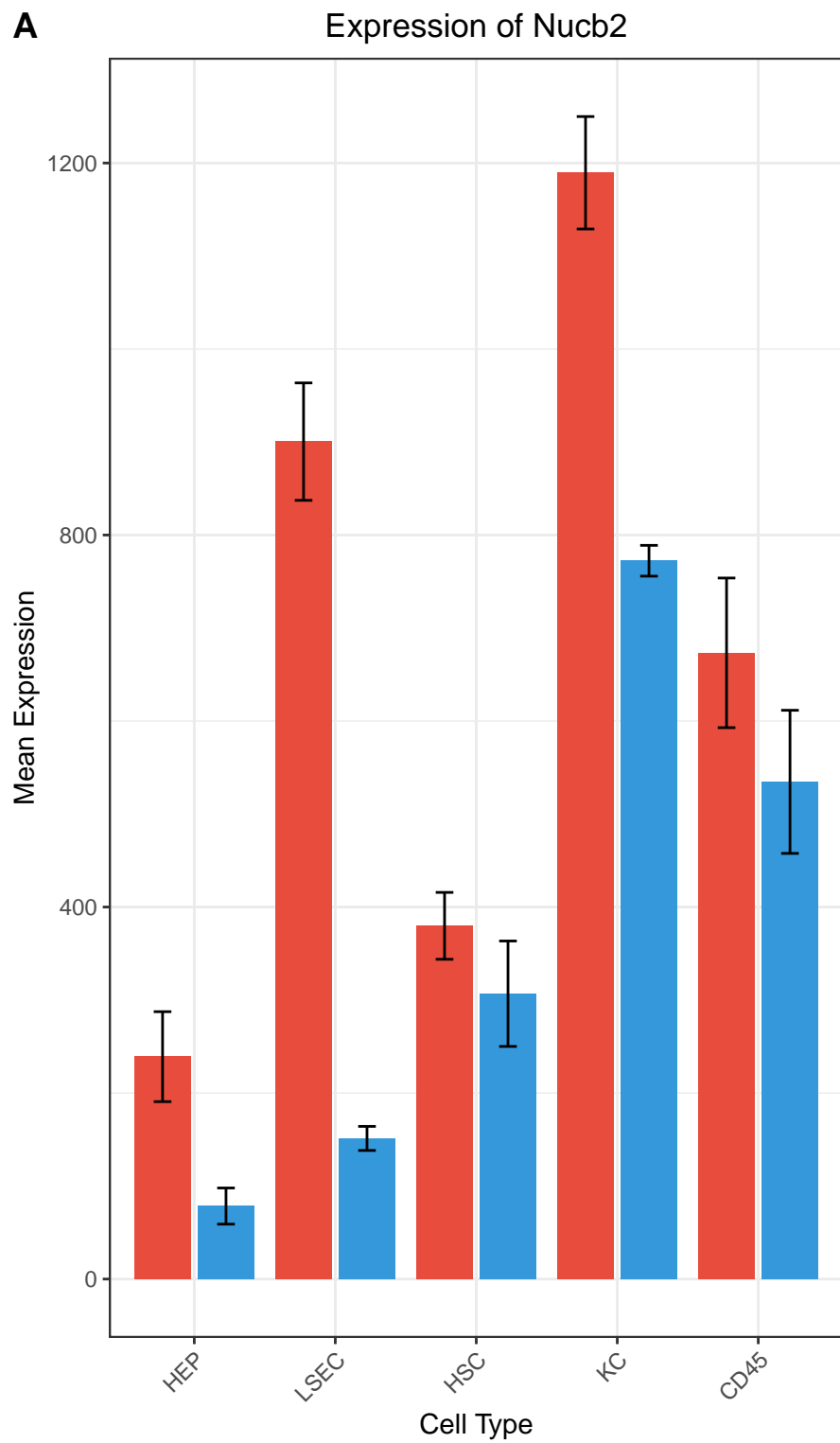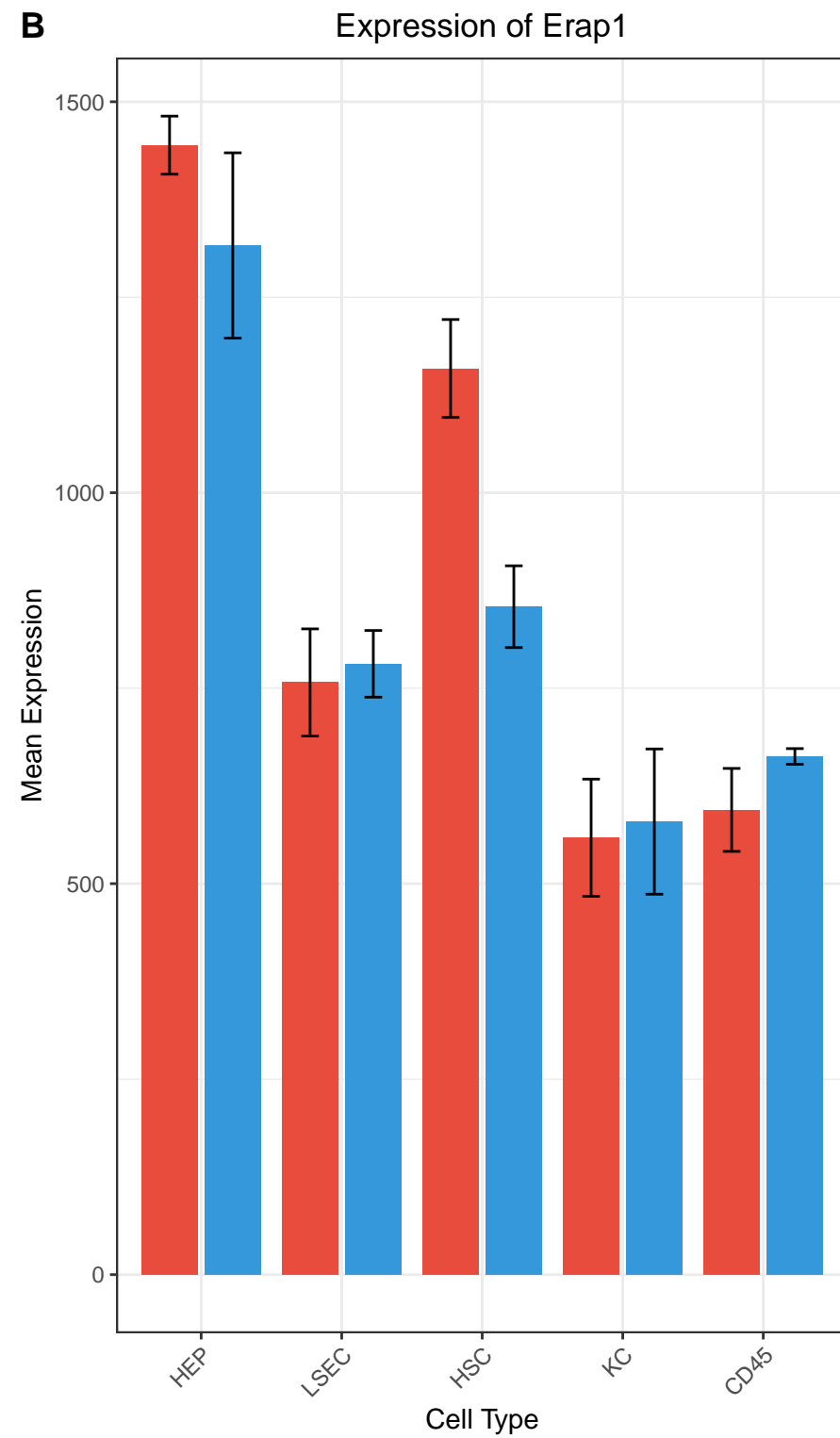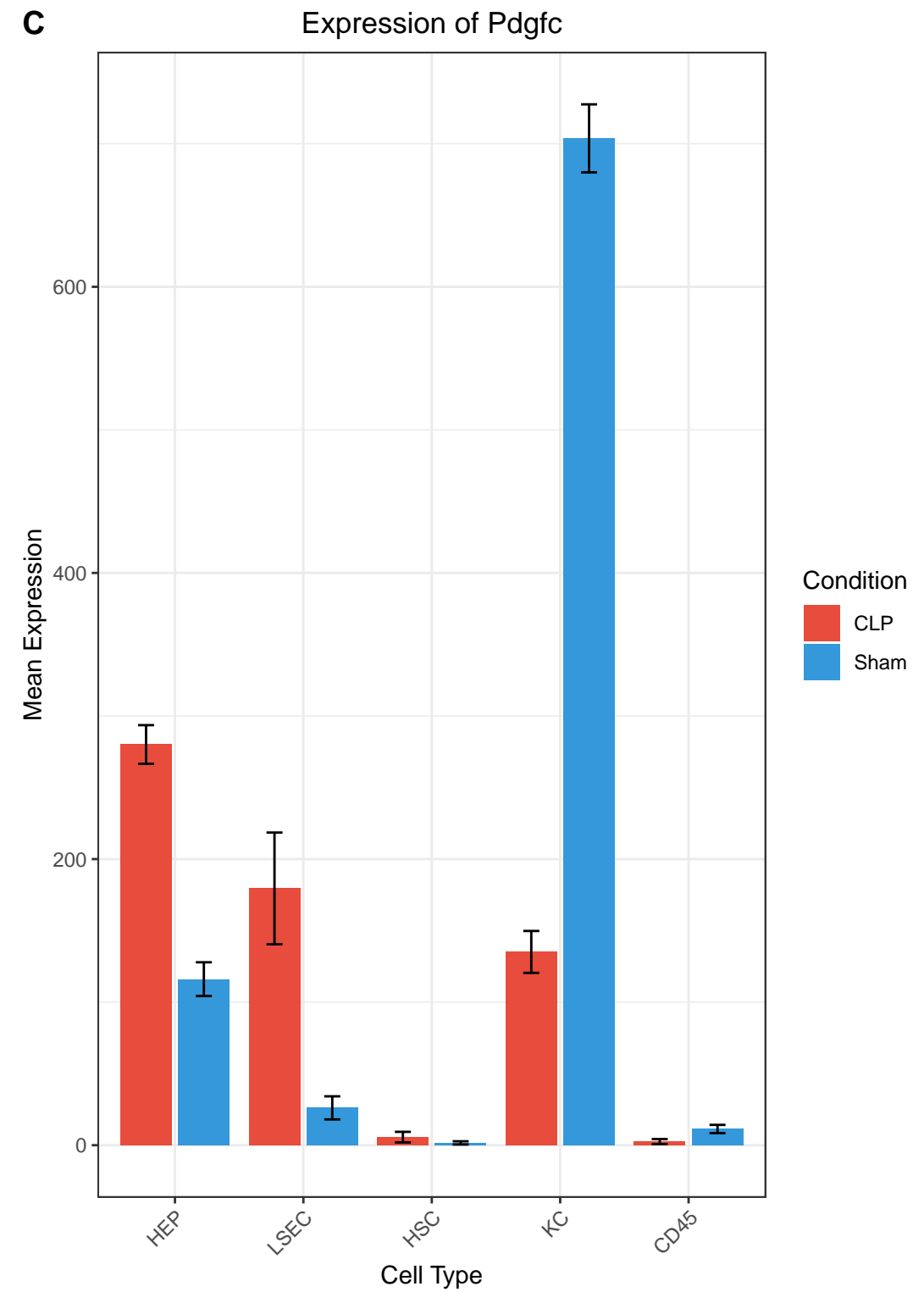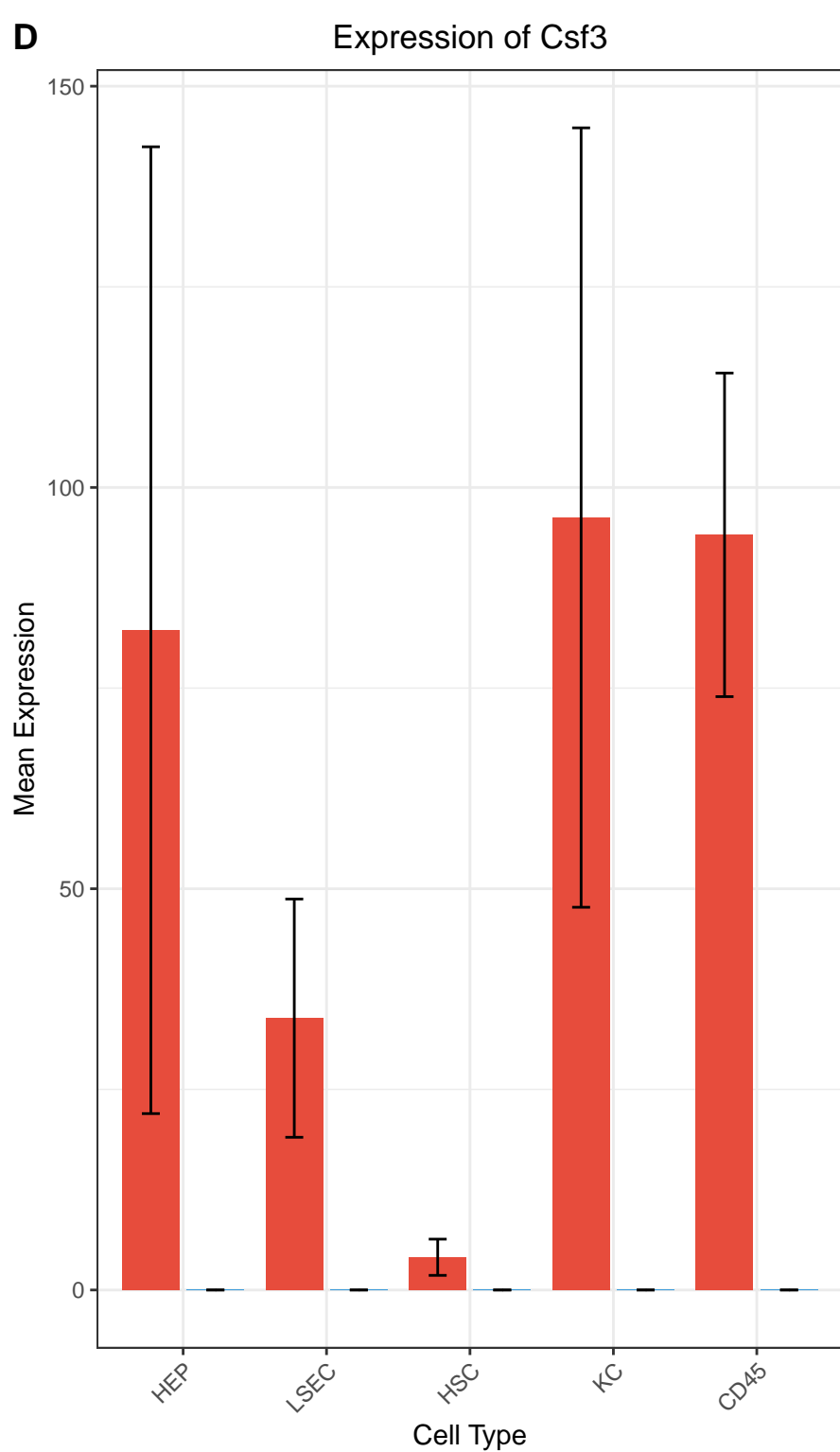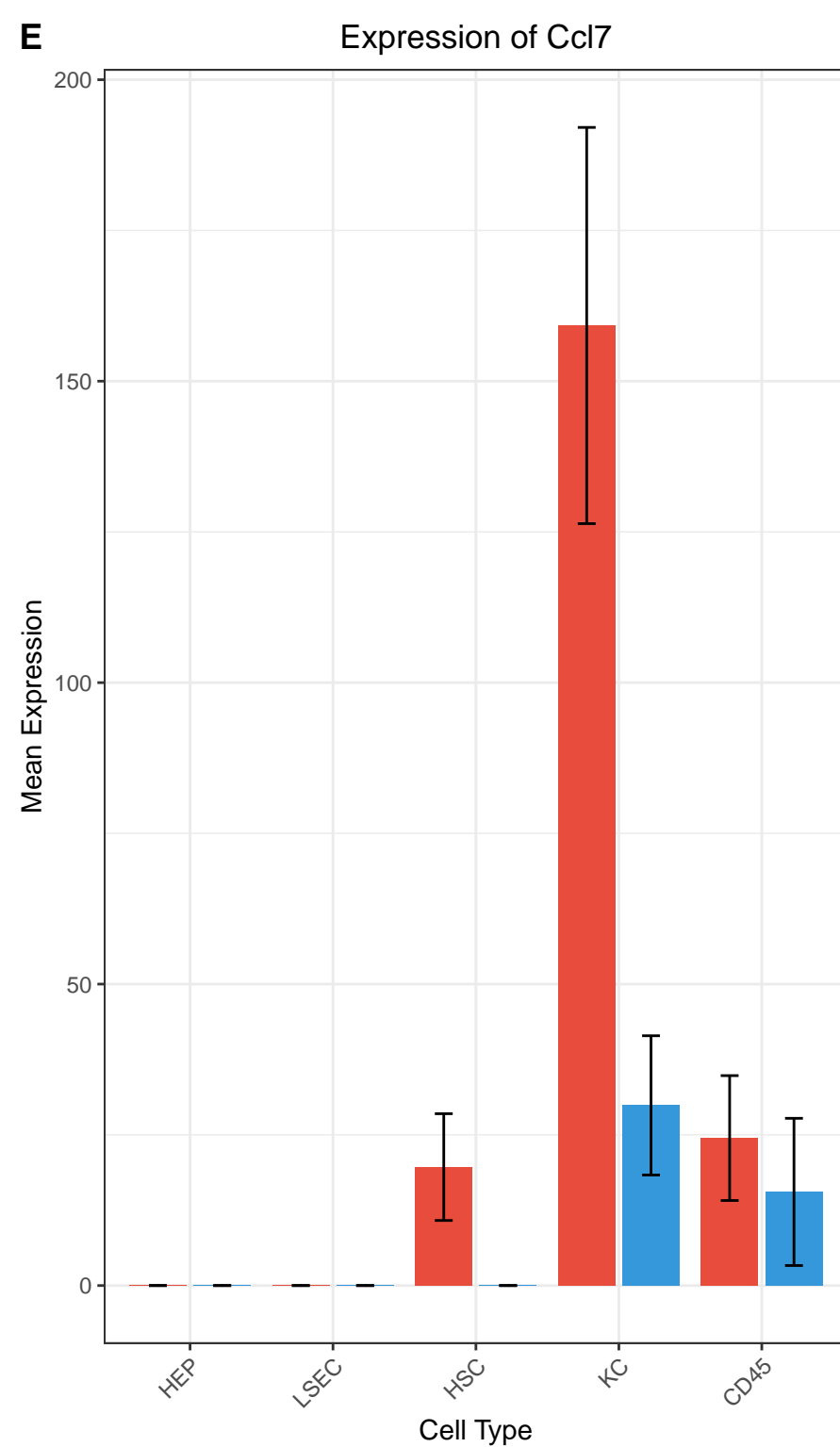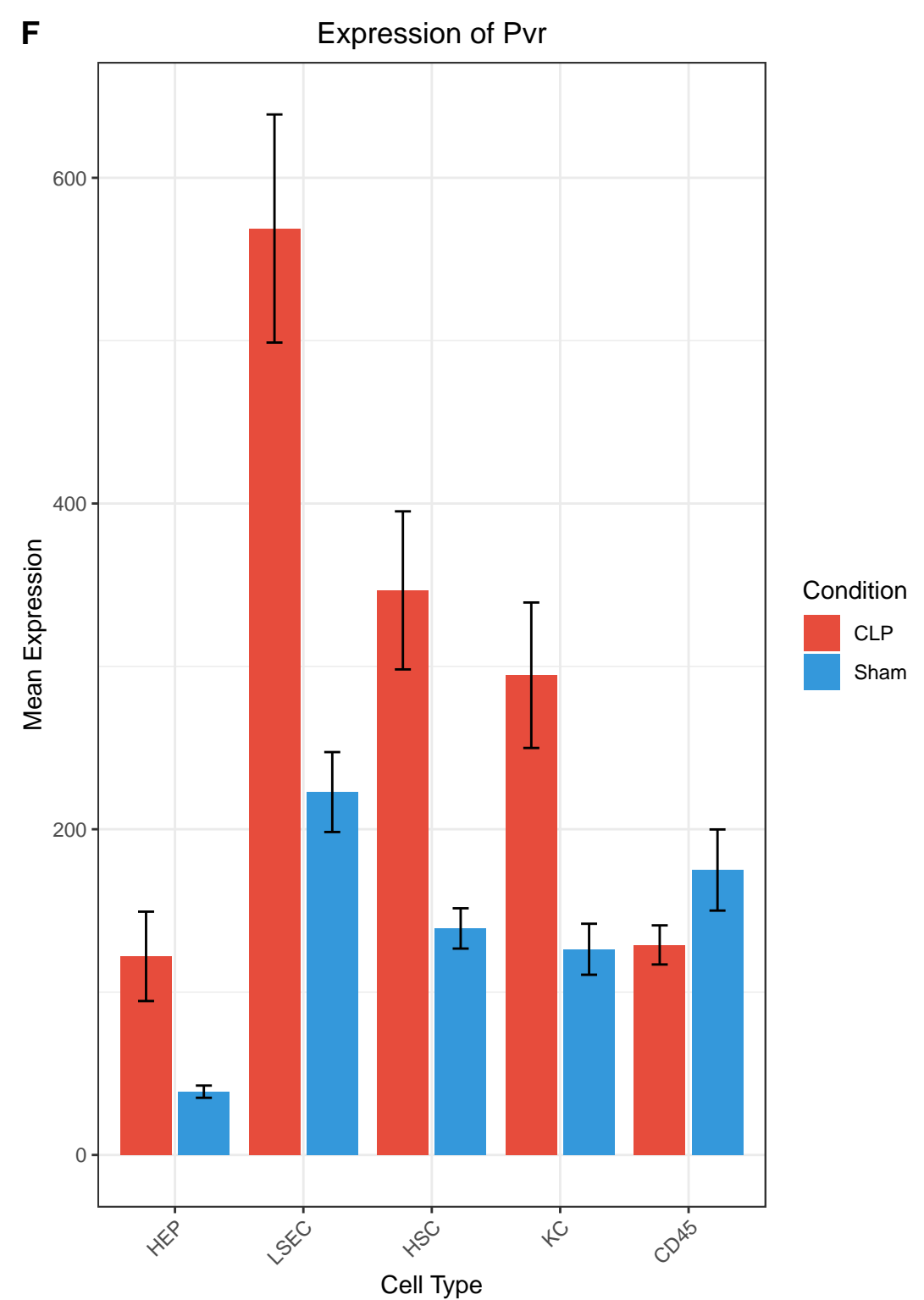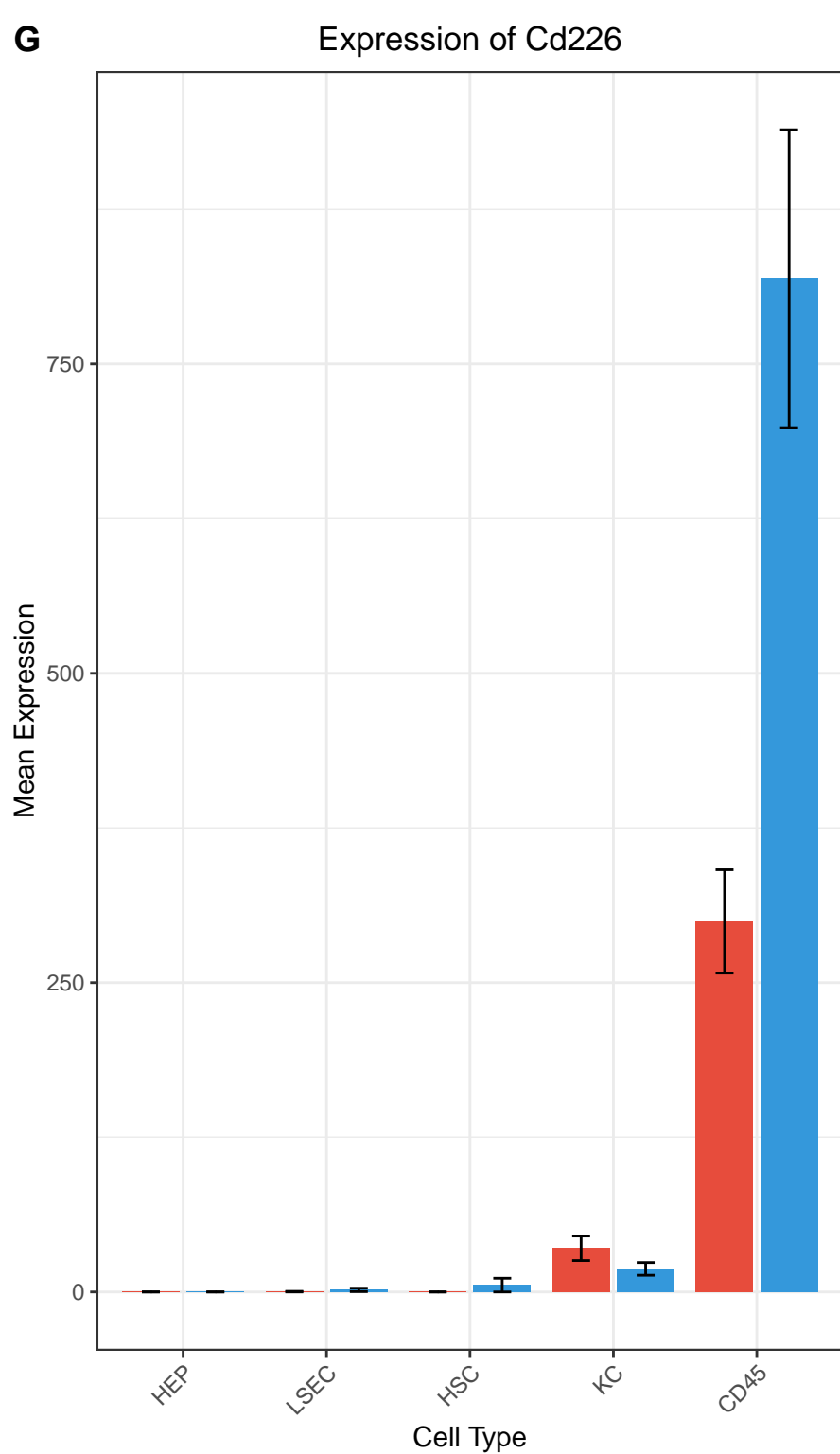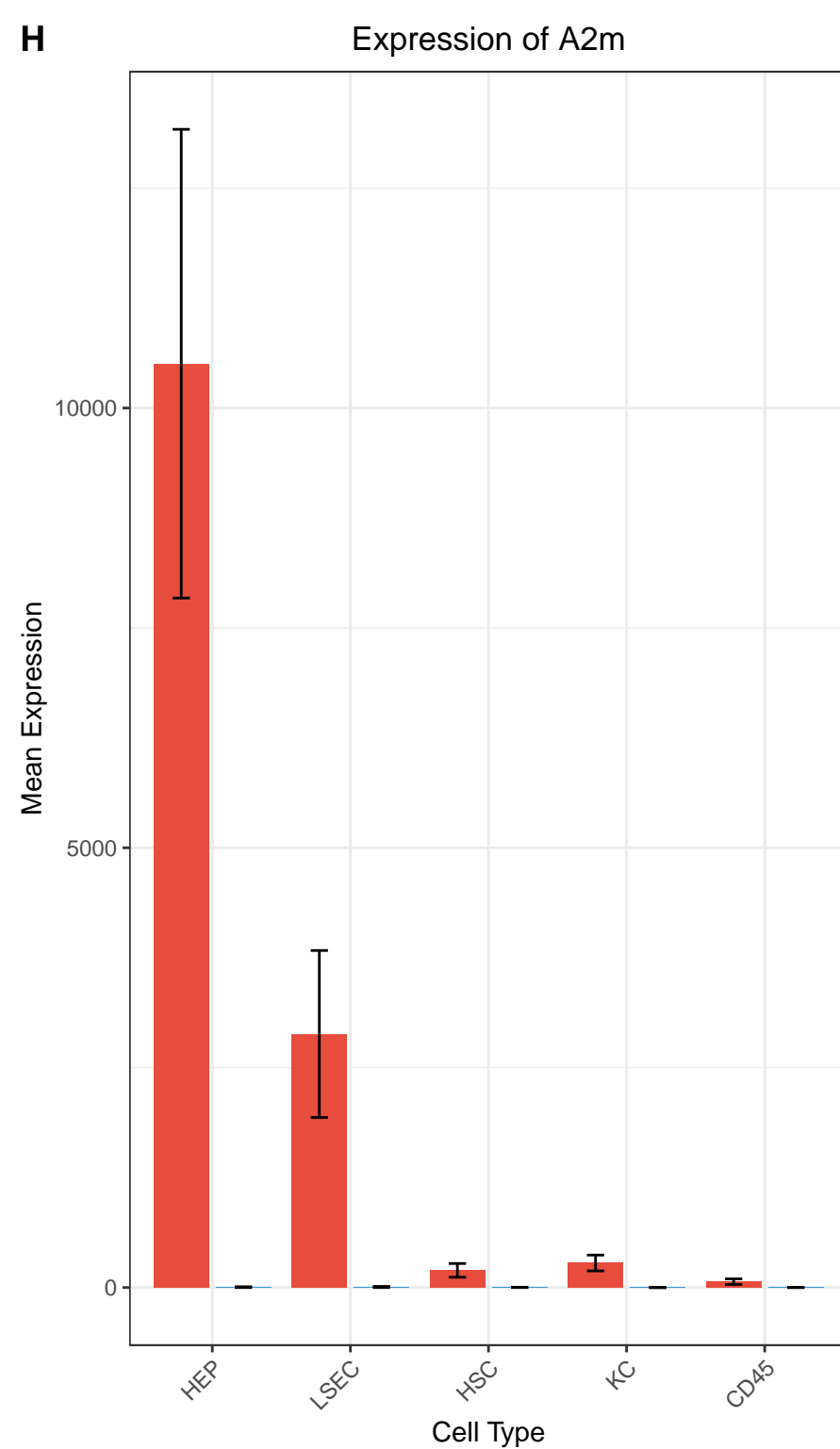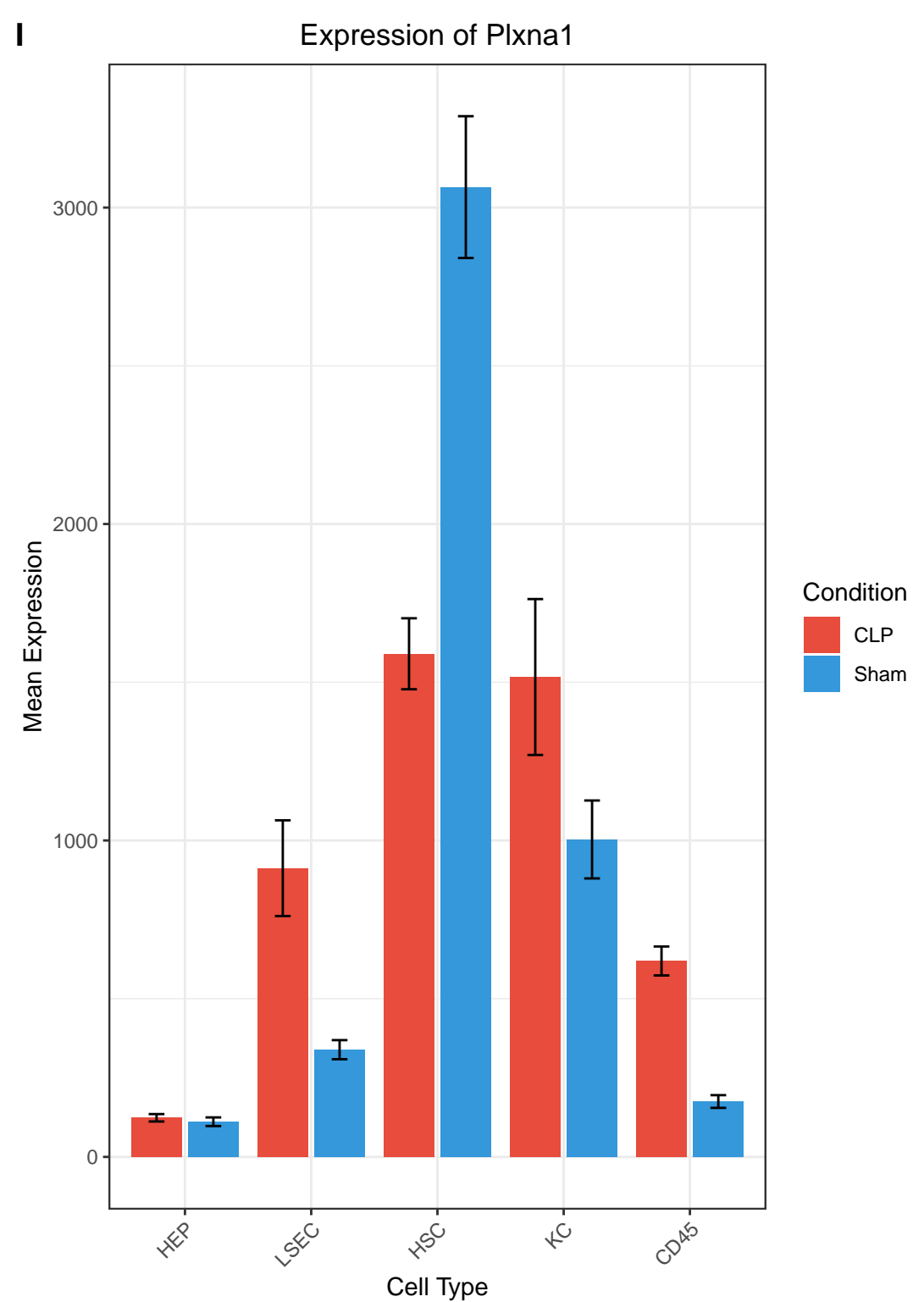

Supplement: Supplementary file 1 [file cells-15-00968-s001.zip › Figure_S8.pdf]
