# Supplementary material for: Polymicrobial Sepsis-Induced Changes in Hepatic Stellate Cell Communication in Male C57BL/6J Mice
Source: Cells. 2026 May 24;15(11):968. doi: 10.3390/cells15110968 (PMC13256657; doi:10.3390/cells15110968)
Supplement: Supplementary file 1 [file Figure_S6.pdf › Figure_S6.pdf]

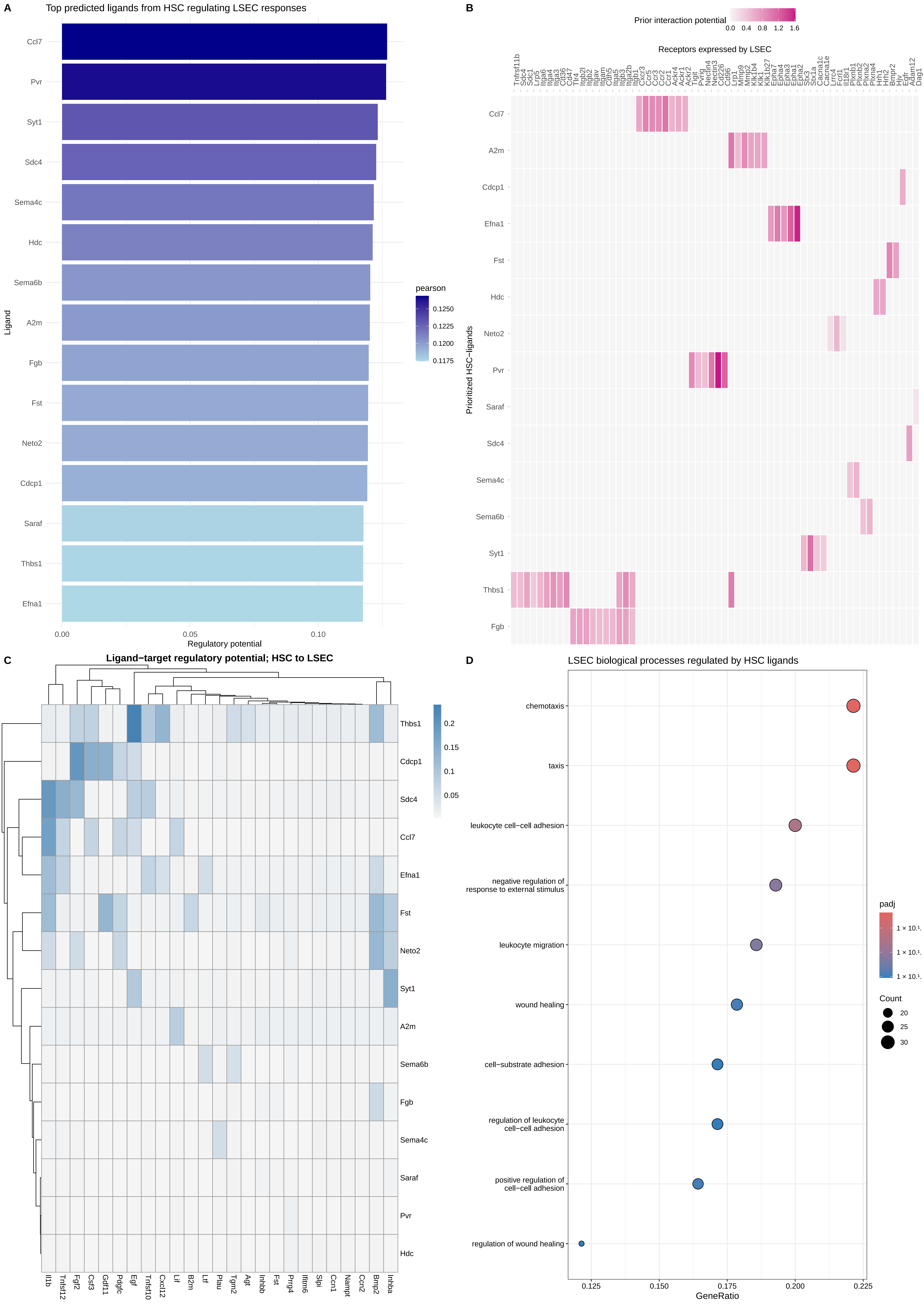
A
Top predicted ligands from HSC regulating LSEC responses
Ligand
Ccl7
Pvr
Syt1
Sdc4
Sema4c
Hdc
Sema6b
A2m
Fgb
Fst
Neto2
Cdcp1
Saraf
Thbs1
Efna1
0.00
0.05
0.10
Regulatory potential
pearson
0.1250
0.1225
0.1200
0.1175
B
Prior interaction potential
0.0 0.4 0.8 1.2 1.6
Receptors expressed by LSEC
Tnfrsf11b Sdc4 Sdc1 Lrp5 Itga6 Itga4 Itga3 Cd36 Cd47 Tlr4 Itgb2l Itgb2 Itgav Itgam Cdh5 Itga5 Itgb3 Itga2b Itgb1 Cxcr3 Ccr5 Ccr3 Ccr2 Ccr1 Ackr4 Ackr1 Ackr2 Tigit Pvrig Nectin4 Nectin3 Cd226 Cd96 Lrp1 Mmp9 Mmp2 Klk1b4 Klk1 Klk1b27 Epha7 Epha4 Epha3 Epha1 Epha2 Stx3 Stx1a Cacna1c Cacna1e Lrrc4 Fcrl1 Il18r1 Plxnb1 Plxnb2 Plxna2 Plxna4 Hrh1 Hrh2 Bmpr2 Hjv Egfr Adam12 Dag1
Prioritized HSC−ligands
Ccl7
A2m
Cdcp1
Efna1
Fst
Hdc
Neto2
Pvr
Saraf
Sdc4
Sema4c
Sema6b
Syt1
Thbs1
Fgb
C
Ligand−target regulatory potential; HSC to LSEC
Thbs1
Cdcp1
Sdc4
Ccl7
Efna1
Fst
Neto2
Syt1
A2m
Sema6b
Fgb
Sema4c
Saraf
Pvr
Hdc
Il1b Tnfsf12 Fgf2 Csf3 Gdf11 Pdgfc Egf Tnfsf10 Cxcl12 Lif B2m Ltf Plau Tgm2 Agt Inhbb Fst Prg4 Ifitm6 Slpi Ccn1 Nampt Ccn2 Bmp2 Inhba
0.2
0.15
0.1
0.05
D
LSEC biological processes regulated by HSC ligands
chemotaxis
taxis
leukocyte cell−cell adhesion
negative regulation of response to external stimulus
leukocyte migration
wound healing
cell−substrate adhesion
regulation of leukocyte cell−cell adhesion
positive regulation of cell−cell adhesion
regulation of wound healing
0.125
0.150
0.175
0.200
0.225
GeneRatio
padj
1 × 10.¹.
1 × 10.¹.
1 × 10.¹.
Count
20
25
30
