# Supplementary material for: Polymicrobial Sepsis-Induced Changes in Hepatic Stellate Cell Communication in Male C57BL/6J Mice
Source: Cells. 2026 May 24;15(11):968. doi: 10.3390/cells15110968 (PMC13256657; doi:10.3390/cells15110968)
Supplement: Supplementary file 1 [file Figure_S7.pdf › Figure_S7.pdf]

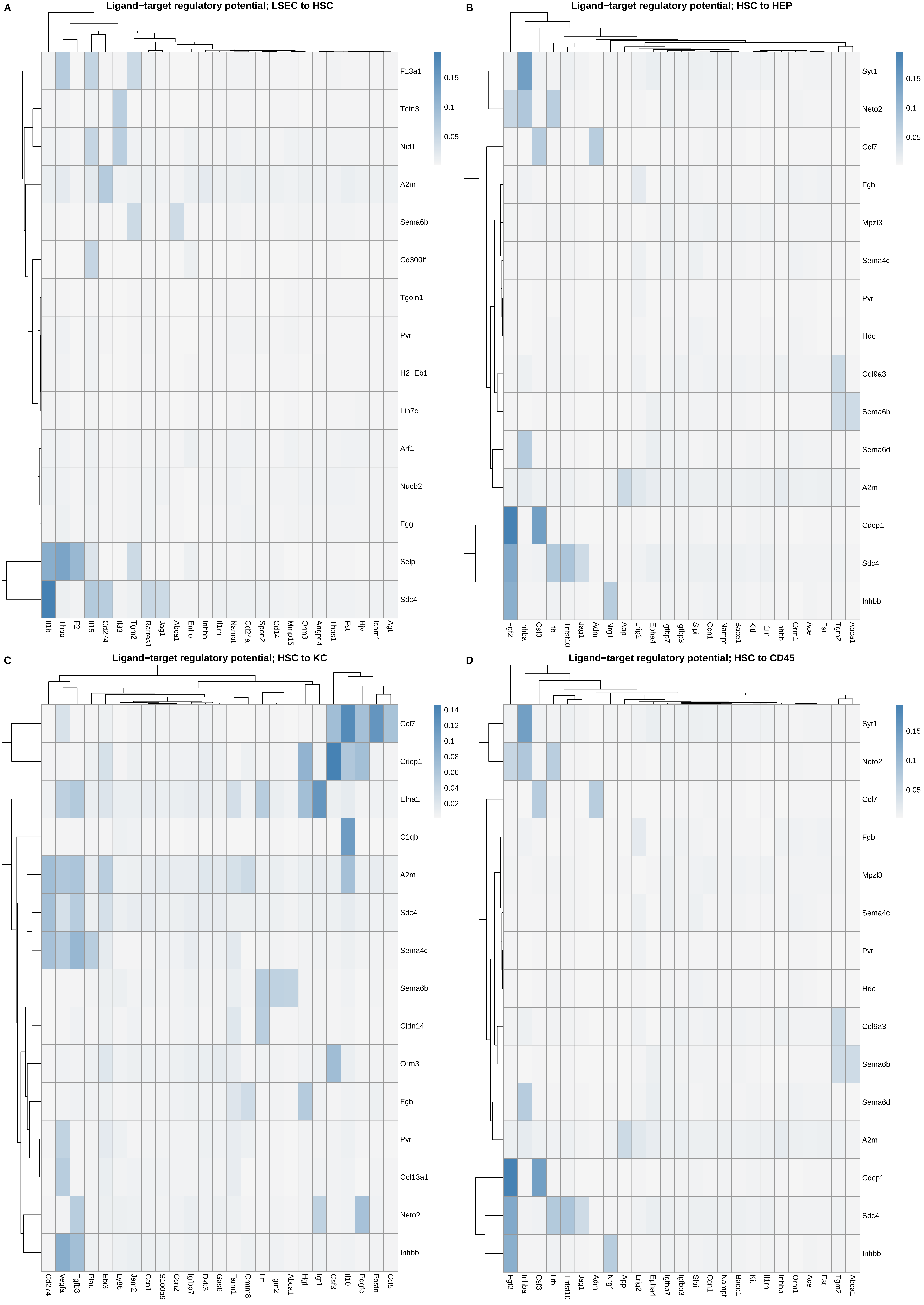
A
Ligand−target regulatory potential; LSEC to HSC
0.15
0.1
0.05
F13a1
Tctn3
Nid1
A2m
Sema6b
Cd300lf
Tgoln1
Pvr
H2−Eb1
Lin7c
Arf1
Nucb2
Fgg
Selp
Sdc4
Il1b
Thpo
F2
Il15
Cd274
Il33
Tgm2
Rarres1
Jag1
Abca1
Efnb
Inhbb
Il1rn
Nampt
Cd24a
Spon2
Cd14
Mmp15
Orm3
Angptl4
Thbs1
Fst
Hjv
Icam1
Agt
B
Ligand−target regulatory potential; HSC to HEP
0.15
0.1
0.05
Syt1
Neto2
Ccl7
Fgb
Mpzl3
Sema4c
Pvr
Hdc
Col9a3
Sema6b
Sema6d
A2m
Cdcp1
Sdc4
Inhbb
Fgf2
Inhba
Csf3
Ltb
Tnfsf10
Jag1
Adm
Nrg1
App
Lrig2
Epha4
Igfbp7
Igfbp3
Slpi
Ccn1
Nampt
Bace1
Kitl
Il1rn
Inhbb
Orm1
Ace
Fst
Tgm2
Abca1
C
Ligand−target regulatory potential; HSC to KC
0.14
0.12
0.1
0.08
0.06
0.04
0.02
Ccl7
Cdcp1
Efna1
C1qb
A2m
Sdc4
Sema4c
Sema6b
Cldn14
Orm3
Fgb
Pvr
Col13a1
Neto2
Inhbb
Cd274
Vegfa
Tgfb3
Plau
Ebi3
Ly86
Jam2
Ccn1
S100a9
Ccn2
Igfbp7
Dkk3
Gas6
Tarm1
Cmtm8
Ltf
Tgm2
Abca1
Hgf
Igf1
Csf3
Il10
Pdgfc
Postn
Ccl5
D
Ligand−target regulatory potential; HSC to CD45
0.15
0.1
0.05
Syt1
Neto2
Ccl7
Fgb
Mpzl3
Sema4c
Pvr
Hdc
Col9a3
Sema6b
Sema6d
A2m
Cdcp1
Sdc4
Inhbb
Fgf2
Inhba
Csf3
Ltb
Tnfsf10
Jag1
Adm
Nrg1
App
Lrig2
Epha4
Igfbp7
Igfbp3
Slpi
Ccn1
Nampt
Bace1
Kitl
Il1rn
Inhbb
Orm1
Ace
Fst
Tgm2
Abca1
